# Uncovering the genomic basis of phenological traits in *Chouardia litardierei* (Asparagaceae) through a genome-wide association study (GWAS)

**DOI:** 10.3389/fpls.2025.1571608

**Published:** 2025-04-17

**Authors:** Sara Laura Šarančić, Nikolina Pleić, Krešimir Križanović, Boštjan Surina, Damjan Mitić, Ivan Radosavljević

**Affiliations:** ^1^ Department of Biology, Faculty of Science, University of Zagreb, Zagreb, Croatia; ^2^ Department of Biology and Human Genetics, School of Medicine, University of Split, Split, Croatia; ^3^ Department of Electronic Systems and Information Processing, Faculty of Electrical Engineering and Computing, University of Zagreb, Zagreb, Croatia; ^4^ Natural History Museum Rijeka, Rijeka, Croatia; ^5^ Faculty of Mathematics, Natural Sciences and Information Technologies, University of Primorska, Koper, Slovenia

**Keywords:** GWAS, local adaptation, adaptive traits, *Chouardia litardierei*, phenology

## Abstract

*Chouardia litardierei* (Asparagaceae) is a non-model, perennial species characterized by exceptional ecological plasticity. In this research, we studied the genetic architecture underlying several phenological traits in selected ecologically diverged populations of this species. We conducted a genome-wide association study (GWAS) to identify genomic regions linked to the following populations-specific phenological traits: Beginning of Sprouting (BOS), Beginning of Flowering (BOF), Flowering Period Duration (FPD), and Vegetation Period Duration (VPD). Combining phenological data from a common garden experiment with an SNP dataset obtained through the ddRAD-seq approach, we identified numerous loci associated with these traits using single- and multi-locus GWAS models. Narrow-sense heritability estimates were high for all traits, with the VPD trait showing the highest estimate (86.95%), emphasizing its importance for local adaptation. Functional annotation of associated genomic regions revealed key protein families involved in flowering time regulation, vegetative growth timing, and stress adaptation. These findings provide insights into the molecular mechanisms of local adaptation in *C. litardierei*’s populations from different habitats, emphasizing the role of genetic factors in phenological trait variation and ecological divergence across populations.

## Introduction

Understanding the genetic basis of phenotypic variation is essential for evolutionary biology, as it elucidates mechanisms underlying speciation, biogeographical distributions, and fitness in natural populations (Savolainen et al., 2013; Mckown et al., 2014). Natural selection acts on allele frequencies within populations, shaping their variation and promoting adaptive traits that enhance survivability and reproductive success (Hu et al., 2020; Walter et al., 2022; Lee et al., 2023). As populations undergo local adaptation, ecological speciation may lead to the emergence of new ecotypes (Turesson, 1922; Todesco et al., 2020) — genetically distinct populations of the same species well-adapted to specific ecological niches (Rundle and Nosil, 2005; Cortés et al., 2018). Although the role of ecotypes in the speciation process remains debated (Lowry, 2012; Fernández-Meirama et al., 2022), several studies highlight their importance in driving genetic divergence along ecological gradients (Lowry et al., 2008; Brandrud et al., 2017; Cortés et al., 2018; Bakhtiari et al., 2019). Rapidly evolving lineages in heterogeneous environments offer valuable insights into the genetic mechanisms driving adaptation and speciation (Feder et al., 2011; Cortés et al., 2018).

Phenology is one of the key features of plants as sessile organisms. It determines the timing of life cycle phases and the duration of growth and reproduction (Schwartz, 2003). Although other factors like photoperiod (Adole et al., 2019; Wang et al., 2020), water availability (Zhou et al., 2024), or selection by pollinators (Sandring and Ågren, 2009) may play an important role as well, temperature is considered to be the environmental element with the most substantial impact on various phenological traits (Schwartz, 2003; Cook et al., 2012). Matching the growth and especially reproduction periods with the optimal environmental conditions is of exceptional evolutionary importance and is strongly influenced by natural selection (Duputié et al., 2015). Among phenological traits, flowering time is particularly sensitive to environmental factors, marking a critical transition from vegetative growth to reproduction (Hill and Li, 2016; Gaudinier and Blackman, 2020). In seasonally variable habitats, where the timing and duration of the vegetational season differ across landscapes, plants must initiate sprouting and flowering within a constrained annual timeframe (Anderson et al., 2012). Therefore, the regulation of flowering time emerges as a frequent target of evolutionary processes (Gaudinier and Blackman, 2020). Ecologically divergent taxa in numerous lineages often have different flowering times (e.g., Heslop-Harrison, 1964; Grant, 1981; Levin, 2000) suggesting that some niche shifts were predicated upon temporal change (Levin, 2006). Consequently, alterations in flowering schedules may allow populations to better exploit different groups of pollinators (e.g., Waser, 1983; Goldblatt and Manning, 1996; Johnson et al., 1998), while movements into new pollinator niches are accompanied by changes in floral attributes (Levin, 2006). Natural selection generally favours bigger individuals at maturity; however, the timing of flowering presents a trade-off between maximizing fecundity and ensuring reproductive completion before adverse conditions, such as drought or winter, occur (Anderson et al., 2012). Species facing water limitations often adjust their flowering phenology to align with peak moisture availability, taking advantage of optimal conditions (Settele et al., 2016). For example, Schmalenbach et al. (2014) found that late-flowering *Arabidopsis* plants coped better with drought by compensating for early growth losses with later recovery, while early-flowering plants, which may flower sooner to exploit available moisture before drought, exhibited lower fitness under the same conditions. High salinity also impairs plant growth in *Arabidopsis*, acting as a suppressive factor that delays flowering time (Li et al., 2007; Lee et al., 2023). Coupled with variation in mating opportunity, temporal variation in sexual phases of individual flowers may have a significant impact on reproductive success in dichogamous plants (Sargent and Roitberg, 2000). Since phenological traits display extensive variations in plants and are often related to local adaptation (Rathcke and Lacey, 1985), the analysis of their genetic background presents a great opportunity to study the mechanisms of the adaptive divergence process.

Investigating the genomic underpinnings of specific traits within the framework of environmental dynamics is essential for uncovering the mechanisms driving local adaptation and elucidating the complex relationship between phenological traits and adaptive responses (Bernatchez et al., 2023). Although much of our understanding of flowering regulation and vegetation duration derives from studies on model organisms such as *A. thaliana* (Engelmann and Purugganan, 2006; Kinmonth-Schultz et al., 2021), significant advancements have also been made in agriculturally important species (e.g., Molla, 2022; Vicentini et al., 2023; Flohr et al., 2017; Song et al., 2023). However, broadening this research beyond model organisms could increase our understanding of the diverse genetic mechanisms governing phenological variation in populations of wild, non-model species facing different ecological pressures in their habitats.

**Figure 1:**
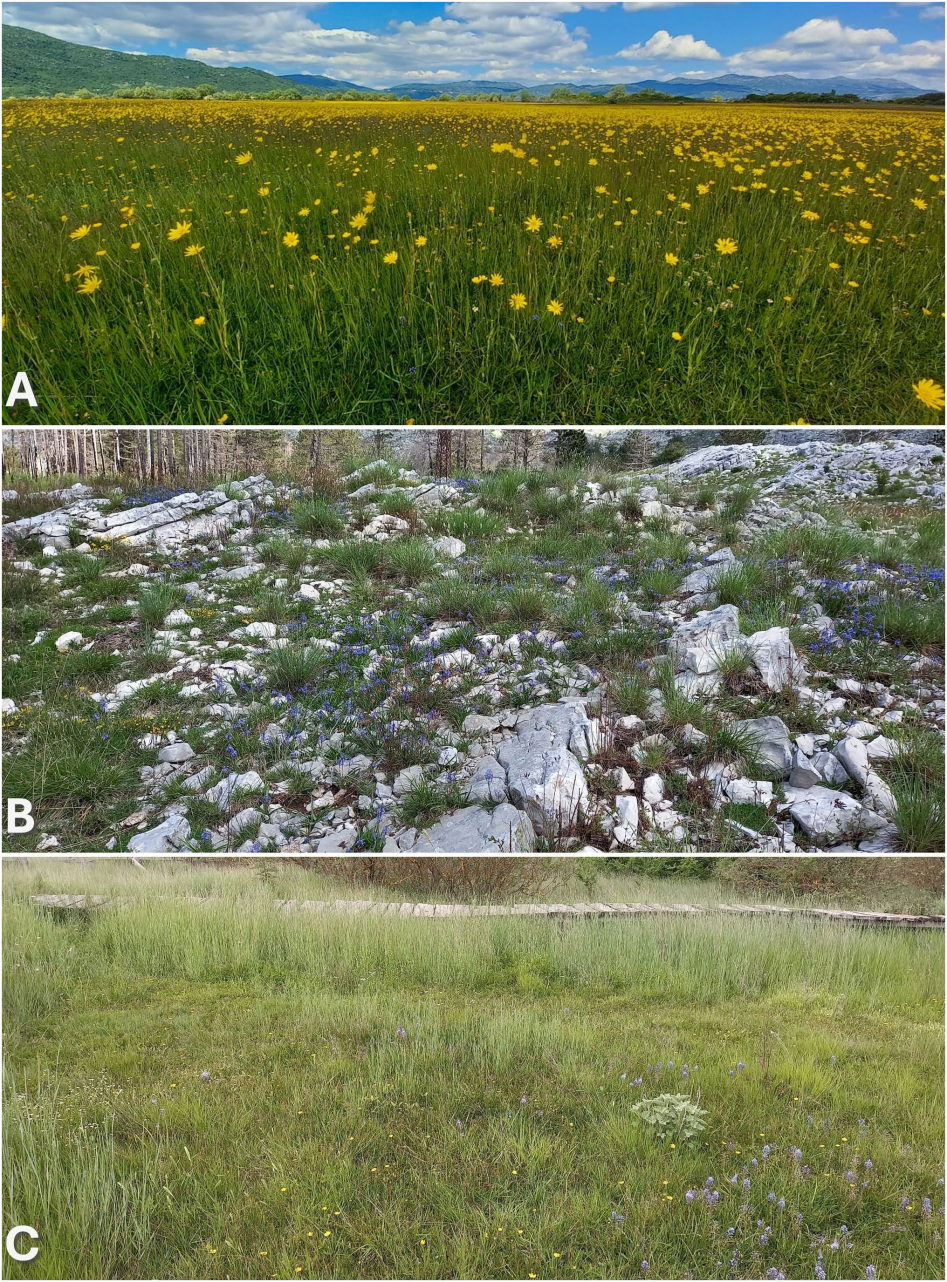
Habitat types of the studied *Chouardia litardierei* populations, shown from top to bottom: **(A)** Karst poljes meadows (locality of Budoške Bare population), **(B)** Dry mountainous grasslands with exposed bedrock (Lovćen), and **(C)** Saline coastal marshes (Vrana Lake).

Here, we investigated the genetic basis of selected phenological traits in the amethyst meadow squill, *Chouardia litardierei* (Breist.) Speta; a small, bulbous, perennial species belonging to the Asparagaceae family [following the APG III system (Bremer et al., 2009)]. Being a typical geophyte, *C. litardierei* plants undergo a dormancy period, which usually stretches from mid-summer to late autumn or early spring, depending on the individual season’s properties. During the spring, soon after the development of young leaves, inflorescence emerges. From late April to early June, depending on the population’s location, the flowering phenophase will unfold, shortly followed by fruiting, which marks the beginning of dying back to an underground perennating organ, i.e., a bulb. *C. litardierei* produces a large racemose inflorescence, typically consisting of several dozen radially symmetrical flowers, without any apparent morphological adaptations for specific pollination mechanisms. While this has not been formally studied, it is expected to be an open-pollinated species (pers. obs.). In addition to sexual reproduction, it propagates clonally through the formation of bulbs surrounding the central bulb. *C. litardierei* populations are found across the Dinaric Alps in the western parts of the Balkan Peninsula (Ritter-Studnička, 1954; Gaži-Baskova, 1962). Throughout this region, populations inhabit highly contrasting habitats, thus indicating a very pronounced ecological plasticity of the species (**Figure 1**).

In terms of habitat types, the most substantial contrast can be observed between southernmost populations, which are found on patches of exposed dolomite bedrocks or dry mountainous grasslands with very thin and sparse soil cover, on one side, and populations occupying lush meadows of karst poljes, enclosed depressions with deep and fertile soils, abundant in water, on another. These groups of populations cope with very different types of challenges. For the first group of the populations, the most substantial adaptation pressure is expected to come from limited resource availability accompanied by pronounced seasonality in water availability and temperature, which are usual for such a habitat (Mota et al., 2021). At the same time, the second group faces seasonal flooding that can last up to seven months each year (Mihevc et al., 2010; Bonacci, 2014). In addition to these two prevailing groups of populations, based on a habitat type, a third and the smallest group can be recognized, the one inhabiting deep-soiled marshes along the coastline in western parts of the species distribution range. These populations situated in proximity to the seashore are experiencing different climates [i.e., Cfa and Csb climate types according to Köppen classification (Köppen, 1918)] than other inland meadow-habitat populations, which, for the most part, are found in habitats characterized by Cfb type of climate. In addition, these seashore populations are exposed to periodical sea flooding, which causes an increase in soil salinity, one of the major factors in plant ecology (Bui, 2013). Nonetheless, it is essential to note that although this issue was already addressed by Šilić (1990), no clear differentiation, either phenotypic or genetic, among these groups of populations has yet been recognized.

To learn as much as possible about the genetic background of phenological traits of selected *C. litardierei* populations from across its distribution range and from different habitats, results from a common garden experiment and genotyping were processed through a set of comprehensive single- and multi-locus genome-wide association (GWA) models. Functional annotation of recognized candidate loci was further performed, thus enabling us to deepen our understanding of the complex genetics behind the phenological aspect of adaptive divergence and to analyze the extent to which differentiation of the studied populations has advanced.

## Methods

### Plant material, common garden experiment, and phenotyping

To establish the common garden experiment, 214 individuals were relocated from nine chosen populations of *C. litardierei*. Three populations were selected to represent each of the three presumed groups of populations from different habitat types, as illustrated in **Figure 1**.

During the sampling expeditions, 22 to 25 individuals were selected from each population, ensuring a minimum distance of 10 meters between them, following the 1:20 rule (Wagner, 1995). The geographic coordinates of the sampling locations are listed in **Supplementary File 1**. Leaf material from each individual was collected for DNA extraction and desiccated using silica gel. Each sampled individual (represented by a single bulb) was transplanted into a separate two-litre plastic container filled with a mixture of soil, sand, and perlite. The containers were placed in raised beds outdoors, creating a common garden setup that exposed the plants to a temperate continental climate (Cfb climate type) (Köppen, 1918; Zaninović et al., 2008). No additional interventions, such as supplemental watering or pesticide use, were applied, allowing the plants to grow under natural, undisturbed environmental conditions.

After two vegetative seasons of acclimatization, four phenological traits were selected for further research (**Table 1**): (i) Flowering Period Duration (FPD), calculated as the number of days from the appearance of the first flower to the last; (ii) Vegetation Period Duration (VPD), measured as the time from sprouting to the opening of the first capsule with ripened seeds, also in days; (iii) Beginning of Flowering (BOF), recorded using the earliest plant flowering dates a reference; and (iv) Beginning of Sprouting (BOS), noted by referencing the sprouting date of the first individual. All traits examined were measured on an individual genotype level and were considered polygenic.

**Table 1:** Descriptive statistics of the *Chouardia litardierei* phenological traits examined in the study.

Overall			
Trait (days)	Description	Median (Q1 – Q3)	Min – max
FPD	Duration from the date of the first to the last flower for each genotype	17 (15 – 18)	9 - 25
VPD	Duration from genotype sprouting to the opening of the first capsule	97 (88 – 107)	55 - 162
BOF	Beginning of flowering considering the flowering date of the first genotype as a reference	11 (10 – 13)	1 - 33
BOS	Beginning of sprouting considering the sprouting date of the first genotype as a reference	56 (52 – 63)	1 - 88

All traits were measured in days. BOF, Beginning of Flowering; BOS, Beginning of Sprouting; FPD, Flowering Period Duration; max, maximum value; min, minimum value; VPD, Vegetation Period Duration; Q1, first quartile; Q3, third quartile.

To assess differences in phenological traits across individual populations and three groups of populations originating from different habitat types, Kruskal-Wallis tests were implemented in the PAST software (Hammer et al., 2001), were performed. We further performed pairwise comparisons using Mann-Whitney *post-hoc* tests with Bonferroni correction to identify significant trait variations. Since none of the variables followed a normal distribution, Spearman’s correlation analysis was conducted to examine the relationships between FPD, VPD, BOF, and BOS variables using the “stats” package in R (R Core Team, 2016).

### Sequencing, genomic data processing, and population genetic structure

DNA isolation was carried out using the GenElute™ Plant Genomic DNA Miniprep Kit (Sigma–Aldrich^®^). DNA concentrations were measured with the Qubit™ Fluorometer (Thermo Fisher Scientific, Wilmington, DE, USA), and samples were subsequently diluted to a concentration of 20 ng/μL.

For genotyping the studied *C. litardierei* populations, a ddRAD-seq approach was utilized (Peterson et al., 2012). DNA was initially digested with two restriction enzymes, AseI and NsiI (NEB #R0526L and #R0127L, respectively). The resulting fragments were then ligated with barcoded i5 and i7 adapters, allowing all samples to be multiplexed. Final amplification was carried out after nick repair using DNA polymerase I (NEB #M0209L). The resulting DNA libraries were double-sequenced (150 bp paired-end) on the Illumina HiSeq X platform.

The initial sequencing data underwent preprocessing for quality trimming and adapter removal using Trim Galore (Martin, 2011). Post-trimming, BAM files were generated by aligning the reads to the *C. litardierei* reference genome (Radosavljević et al., 2023) using the Burrows-Wheeler Aligner (Li and Durbin, 2009). SNP identification was performed with the Stacks software package v1.48 (Catchen et al., 2013). The ref_map.pl wrapper module was utilized, following Paris et al. (2017) recommendations, the *pstacks* module was executed to extract loci previously aligned to the reference genome, with a minimum coverage depth of three reads to ensure a reliable representation of loci across samples and reduce low-confidence genotype calls. The *cstacks* module then constructed a comprehensive catalogue of loci across populations, allowing a maximum of four mismatches among sample loci to minimize alignment errors. Subsequently, the *populations* module calculated population-level summary statistics. To ensure high data quality, loci were retained only if present in all nine populations and at least 70% of individuals within each population, with a maximum observed heterozygosity of 0.70. Additional filtering criteria included retaining only one SNP per locus and excluding loci with minor allele frequencies (MAF) below 1%. This stringent filtering approach focused on common and well-represented genetic variants, reducing the risk of inaccuracies due to sequencing or sampling errors. The resulting dataset, comprising high-quality genetic markers, was exported in .vcf format for downstream analysis.

To assess the neutral population genetic structure of the studied populations, we used a model-based clustering method implemented in ParallelStructure (Pritchard et al., 2000; Besnier and Glover, 2013). To overcome the issue of this analysis’s high computational demands and lengthy processing time for such a large number of SNPs, we constructed a subset of 5,000 randomly selected SNPs. The analysis comprised ten runs for each of the ten clusters (K). Each run consisted of a burn-in period of 50,000 steps, followed by 500,000 Monte Carlo Markov Chain (MCMC) replicates. We used the StructureSelector online software (Li and Liu, 2018) to obtain the most likely number of clusters (K) following Evanno’s method (Evanno et al., 2005) as well as to retrieve the final data through the clustering and averaging of the runs following the Clumpak algorithm (Kopelman et al., 2015). The obtained results were processed using CorelDRAW X7 v.17.1.0.572 software (Corel Corp., Ottawa, Canada) for improved visualization.

### Genome-wide association analyses

**Figure 2:**
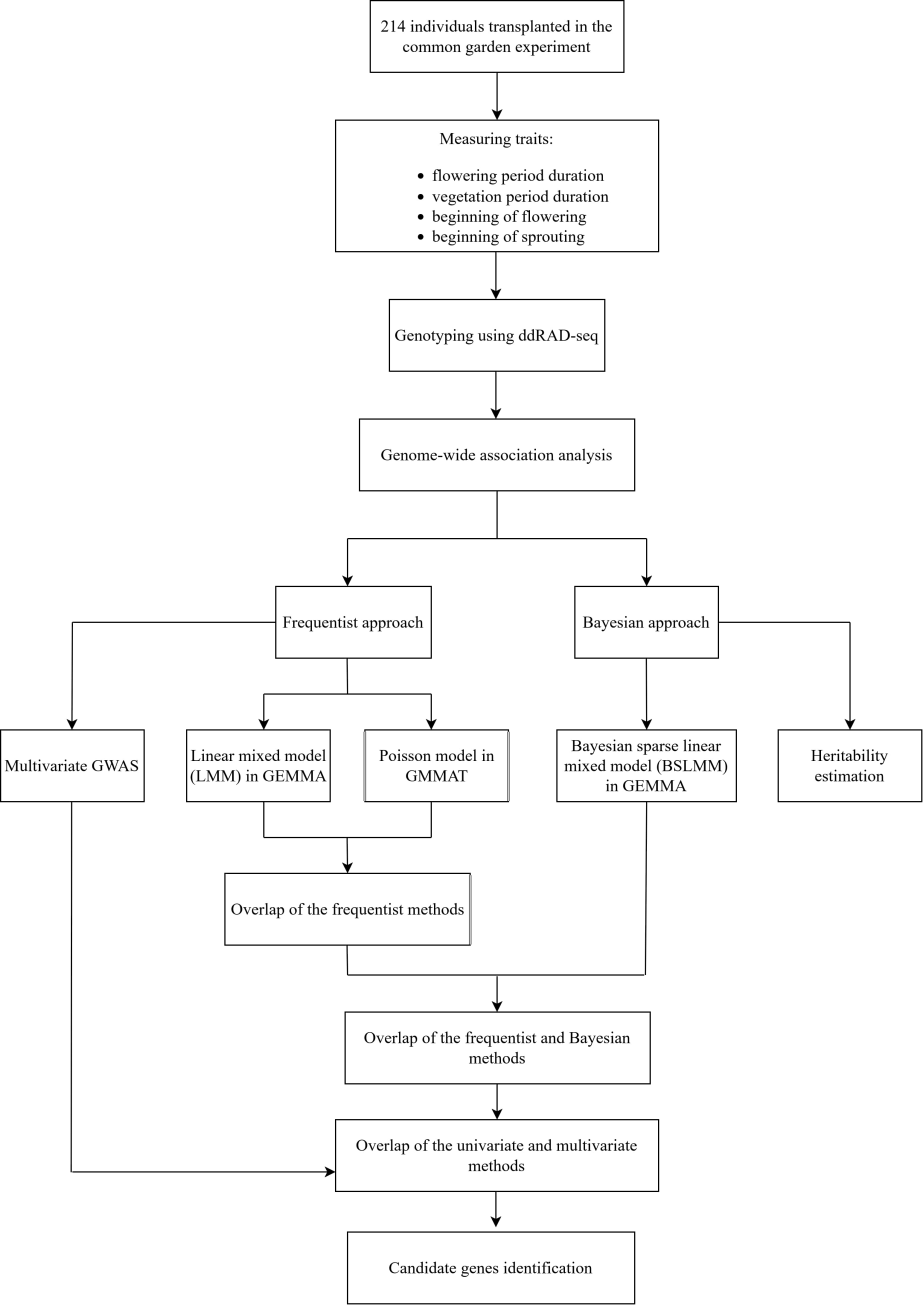
A schematic outline of the methodological approach employed to study the genetic basis of phenological traits in *Chouardia litardierei*.

**Figure 2** illustrates a schematic representation of the methodological approach used in this study. All traits were treated as polygenic and GWAS analyses were carried out assuming an additive genetic model. Variants with a minor allele frequency (MAF) below 1% were excluded using the BCFtools software (Danecek et al., 2021). Two distinct statistical approaches were employed for each association analysis: the frequentist single-locus approach and the Bayesian multi-locus approach. In the frequentist single-locus approach, two distinct models were applied. A standard linear mixed model (LMM) was fitted using GEMMA 0.98.5 (Zhou and Stephens, 2012) for all four traits, keeping in mind that this approach assumes a normal trait distribution. Additionally, all traits were analyzed using GMMAT 1.4.2 (Chen et al., 2019), applying a Poisson generalized linear mixed model (GLMM), to account for their count-based distributions. The Poisson GLMM in GMMAT was selected because it effectively accounts for the non-normal distribution of count data, providing a complementary approach to the LMM analysis performed in GEMMA.

In the Bayesian multi-locus approach, a Bayesian sparse linear mixed model (BSLMM) (Zhou et al., 2013) was simultaneously fitted for all traits under analysis. Significant SNPs for each trait were identified by first intersecting the sets of significant SNPs obtained from GLMM and LMM, and then further intersecting the resulting set with those identified by BSLMM, ensuring consistency across both the frequentist and Bayesian approaches (**Figure 2**). Additionally, a multivariate linear mixed model (mvLMM) was performed in GEMMA to simultaneously analyze significantly correlated traits (FPD and VPD, as well as BOF and VPD) to identify shared association signals between them.

The results were visualized using Manhattan plots generated with the R package “qqman” (Turner, 2018) and “CM plot” (Yin et al., 2021). An *ad hoc* threshold of 1×10^−^³ was used for the frequentist GWAS analyses (GLMM, LMM, and mvLMM).

### Generalized linear mixed model using a poisson distribution

The generalized linear mixed model (GLMM) with a Poisson distribution was applied using GMMAT, and the model is expressed as follows (Equations 1–3):


(1) $$log\left(\mu_{i}\right)=W_{i}\alpha +x_{i}\beta +u_{i}$$

(2) $$u∼MVN_{n}\left(0,\;\lambda K\right)$$

(3) $$y_{i}∼Poisson(\mu_{i})$$

In this model, *y_i_* ​ represents the observed count for the *i*-th individual, while *μ_i_* denotes the mean count, modeled as the exponential of the linear predictor. ​**W_i_** is the *i*-th row of an *n × c* matrix of covariates (fixed effects), *α* is the corresponding vector of coefficients for these covariates, **x_i_** represents the genotype of the *i*-th individual, and *β* denotes the effect size of the genetic marker. The random effects *u* are assumed to follow a multivariate normal distribution MVN*_n_* (0,λK), where **K** is the relatedness matrix of size *n × n*, and λ represents the ratio of variance components. The observed data *y_i_* ​ is assumed to follow a Poisson distribution with *μ_i_* ​. This model incorporates individual-level random effects and a genetic relationship matrix **K** to account for population structure and relatedness. When assuming a normal distribution and an identity link function for continuous traits, GMMAT conducts association tests using linear mixed models (LMMs).

### Linear mixed model

The standard LMM was applied using GEMMA 0.98.5. in the following form:


(4) y=Wα+xβ+u+ ϵ

(5) $$u∼MVN_{n}\left(0,\;\lambda \tau^{-1}K\right)$$

(6) $$\epsilon ∼MVN_{n}\left(0,\;\tau^{-1}I_{n}\right)$$

Here, **y** represents a vector of trait values for 214 individuals, and **W** is an *n × c* matrix of covariates (fixed effects), which, in this case, consists of a column of 1s. Let *α* represent a c-vector of the intercept, **x** be an *n*-vector of marker genotypes, and *β* denote the effect size of the marker. Additionally, **u** is an *n*-vector of random effects, *ϵ* is an *n*-vector of errors, *τ* ^-1^ denotes the variance of the residual errors, and *λ* is the ratio between the two variance components. **K** is the known *n × n* relatedness matrix, and **I**_n_ is an *n × n* identity matrix. *MVN_n_* refers to the *n*-dimensional multivariate normal distribution. The effect sizes indicate the change in trait values associated with each additional effect allele in the genotypes of individuals.

### Bayesian framework

The LMM (Equations 4–6) implemented in GEMMA evaluates the alternative hypothesis *H*_1_: *β* ≠ 0 against the null hypothesis *H*_0_: *β* = 0 for each SNP individually. Extensions of the LMM that account for the effects of variants across multiple loci simultaneously could improve the power to identify causal variants. Bayesian LMMs can model all markers simultaneously by assigning different prior distributions to the marker effects and sampling from their posterior distribution. These Bayesian models, designed for estimating SNP effect sizes, start with a basic linear model that links genotypes **X** to phenotypes **y**:


(7) $$y=1_{n}µ+X\beta +\epsilon$$

(8) $$\epsilon ∼MVN_{n}\left(0,\;\tau -1I_{n}\right)$$

we let **y** be a vector of phenotypes observed on *n* individuals, and **X** be an *n × p* matrix of genotypes for these same *n* individuals at *p* genetic markers. The vector *β* represents the effects of genetic markers, **1_n_** is an *n*-vector of 1s, *µ* is a scalar representing the mean phenotype, and *ϵ* is an *n*-vector of error terms with variance *τ* ^-1^. Our aim was to estimate the parameter *β*, which corresponds to the effects of the genetic markers. However, because the number of genetic markers in our study (*p =* 23,315) far exceeds the number of individuals (*n =* 214), certain modeling assumptions regarding SNP effect sizes *β* had to be made. These assumptions range from the infinitesimal (or polygenic) model, which posits that all SNPs have non-zero effects, to the sparse model, which assumes that only a small subset of SNPs affect the phenotype. The success of the model relies on the true genetic architecture of the trait being studied, although this is typically unknown. The most widely used polygenic model assumes that all SNPs impact the phenotype (i.e., have non-zero effects) with normally distributed effect sizes:


(9) $$\beta ∼N(0,\sigma_{\beta }^{2})$$

When Equations 7–8 are combined with the normality assumption (Equation 9) for effect sizes b, they result in the previously described LMM, as it incorporates a random effect term that represents the combined genetic effects.

### Bayesian sparse linear mixed model

A more general assumption, which includes both polygenic and sparse modeling scenarios, suggests that effect sizes come from a mixture of two normal distributions.


(10) $$\beta_{i}\;∼\;\pi \text{N}(0,\frac{\sigma_{a}^{2}+\;\sigma_{b}^{2}}{\text{pτ}})+\left(1-\pi \right)\text{N}(0,\frac{\sigma_{b}^{2}}{\text{pτ}})$$

In this model, π represents the proportion of SNPs with large effects, while σ^2^_β_ and σ^2^_α_ correspond to the variances of small and large effects, respectively. The resulting BSLMM model combines polygenic and sparse effects in the prior distribution of effect sizes, allowing it to adapt to various genetic architectures of the traits being studied. BSLMM addresses population structure and relatedness by incorporating a genomic kinship matrix as a random effect term, and it accounts for linkage disequilibrium (LD) by estimating SNP effect sizes *β* while controlling for other SNPs in the model. The model uses a Markov chain Monte Carlo algorithm to sample from the posterior distribution and estimate SNP effect sizes. Unlike LMM, which provides *p*-values, BSLMM outputs a posterior inclusion probability (PIP) for each SNP, reflecting the likelihood that a marker is associated with the trait based on the data. This PIP is calculated as the proportion of chain iterations in which the SNP exhibits a large effect. SNPs with high PIPs are considered the most likely functional variants influencing the analyzed traits. We applied BSLMM to the same dataset (214 individuals and 23,315 variants) used in our primary frequentist association analysis to compare single-SNP and multi-SNP approaches and reduce false positives. The BSLMM chain was run with 1,000,000 sampling steps and 100,000 burn-in iterations. We used the estimated PIPs from BSLMM for additional fine-mapping of genomic regions identified in the frequentist analysis.

### SNP heritability estimation

The proportion of variance in phenotypes accounted for by all available genotypes (PVE), also referred to as narrow-sense heritability (h^2^), along with the proportion of genetic variance explained by variants with large effects (PGE), was estimated for the traits shown in **Table 1**. This estimation was based on the assumption that SNP effect sizes follow a mixture of two normal distributions (Equation 10), as implemented in GEMMA BSLMM.

### Multivariate genome-wide association analyses

To identify common variants associated with the trait pairs showing the strongest statistically significant correlations, multivariate genome-wide association analyses were performed using a multivariate linear mixed model (mvLMM) in GEMMA. Specifically, multivariate GWAS was conducted for the VPD and BOS traits, as well as for the VPD and BOF traits, which exhibited the strongest statisticaly significant correlations. This approach enabled the simultaneous analysis of genetic effects on both trait pairs of traits by treating them as dependent variables. The mvLMM method accounts for population structure and relatedness among individuals, ensuring accurate identification of genetic variants contributing to the observed phenotypic variation in these traits.

### Candidate genes prediction

After identifying phenotypic evidence for local adaptation in distinct *C. litardierei* populations and conducting GWAS analysis, efforts focused on pinpointing associated candidate genes. Using the reference genome, sequences were extracted spanning a total of 50 kilobases – including 25 kilobases upstream and downstream of each significant SNP identified through both statistical models, using SAMtools (Danecek et al., 2021). Functional annotations for these sequences were then obtained through the eggNOG-mapper v2 database, applying an *e*-value threshold of < 1 × 10^−2^ (Huerta-Cepas et al., 2019).

## Results

### Phenotyping

**Figure 3:**
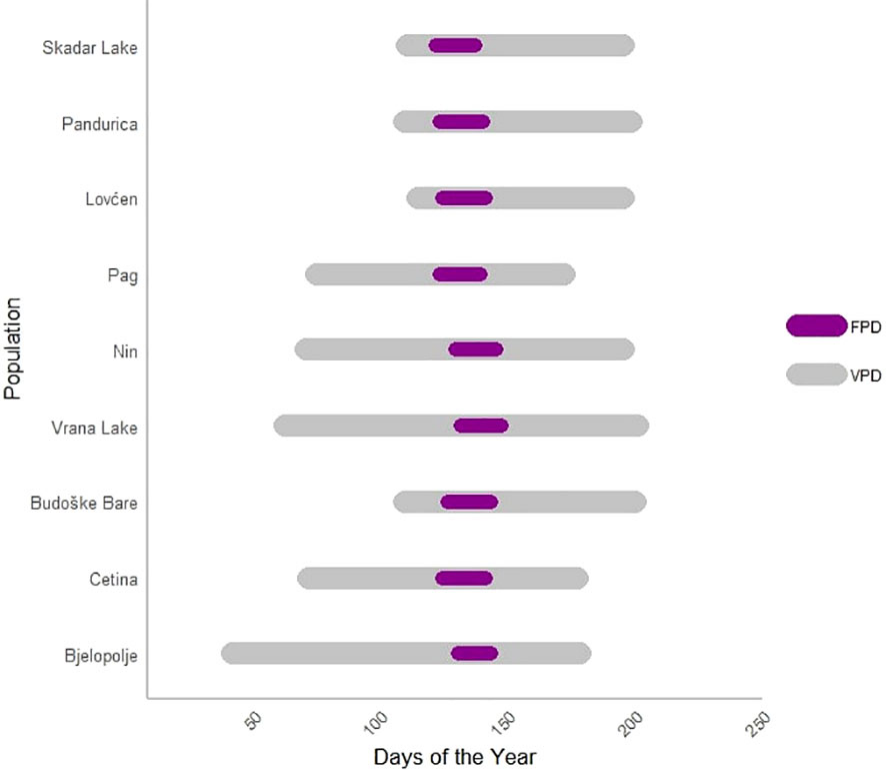
The horizontal bar plot illustrates the durations of Vegetation period Duration (VPD) and Flowering Period (FPD) across populations of the *Chouardia litardierei* during one vegetational season. The x-axis represents the days of the year, while the y-axis lists the populations being compared. The dark magenta bars indicate the FPD, which represents the duration from the date of the first to the last flower for each genotype. In contrast, the grey bars represent the VPD, denoting the duration from the genotype sprouting to the opening of the first capsule. Additionally, the figure provides a visual reference for the Beginning of Flowering (BOF) and the Beginning of Sprouting (BOS), where BOF and BOS are calculated relative to the individual that flowered or sprouted first, respectively.

**Figure 3** illustrates the phenological variations observed among *C. litardierei* populations in the common garden experiment.

**Figure 4:**
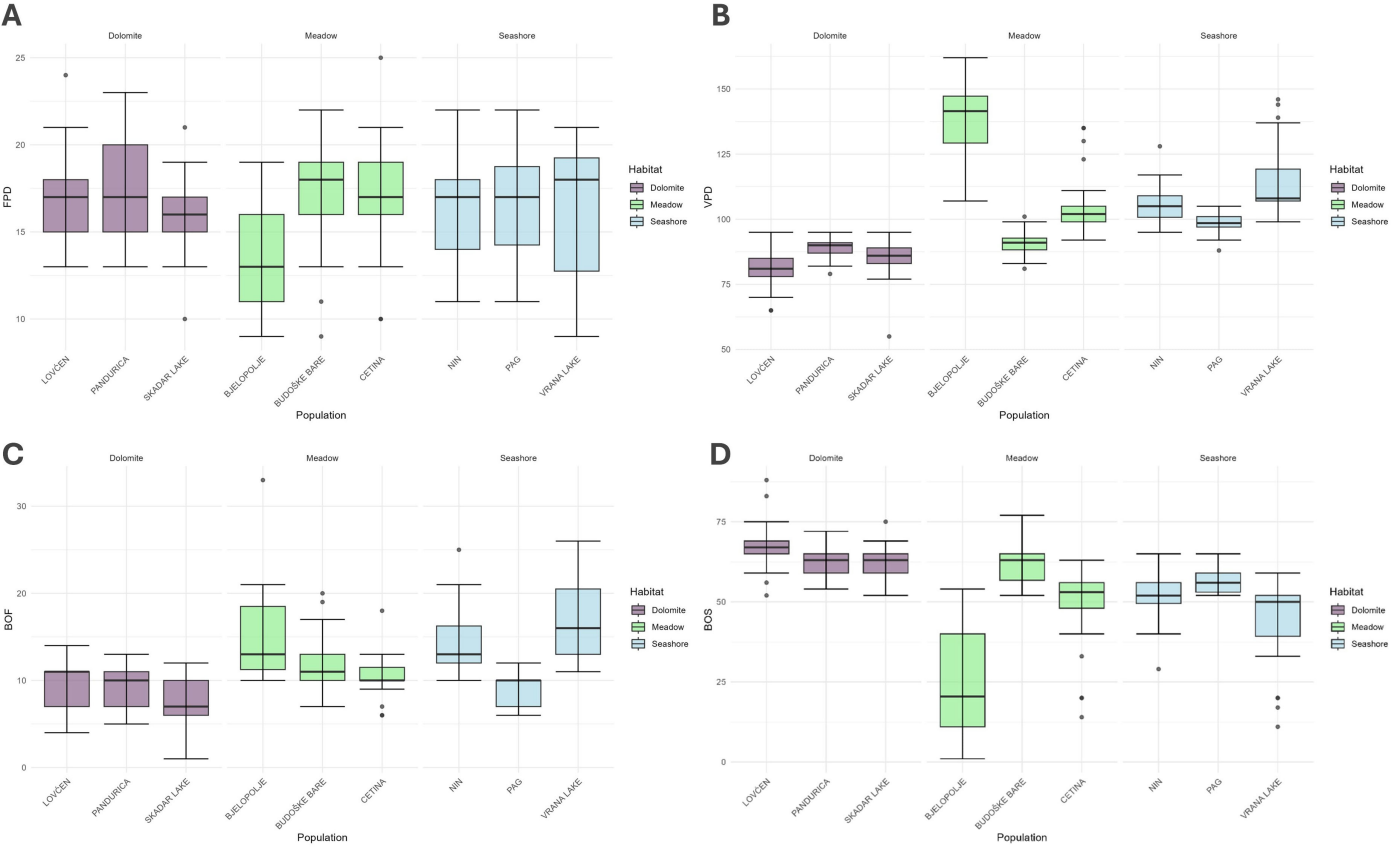
Box plots illustrate the obtained phenological results from a common garden experiment, depicting four phenological traits: **(A)** Flowering Period Duration (FPD) (top left), **(B)** Vegetation Period Duration (VPD) (top right), **(C)** Beginning of Flowering (BOF) (bottom left), and **(D)** Beginning of Sprouting (BOS) (bottom right) per genotype. Each box represents the interquartile range (IQR), with the horizontal line inside the box indicating the median. Whiskers extend to data points within 1.5 times the IQR, while dots represent outliers.

Out of the 214 individuals sampled across nine populations, 204 flowered successfully. Consequently, all traits related to flowering [FPD, VPD (since its ending is related to the start of the fruiting phenophase), and BOF] were measured and subsequent analyses were performed on the set of 204 individuals, while the remaining 10 were discarded. At the same time, the BOS trait was analyzed across all 214 individuals. The FPD and the VPD ranged from 9 to 25 days, with a median of 17 days (Q1 – Q3: 15 – 18), and 55 to 162 days, with a median of 97 days (Q1 – Q3: 88 – 107), respectively. The BOF and BOS traits ranged from 1 to 33 days, with a median of 11 days (Q1 – Q3: 10 – 13), and 1 to 88 days, with a median of 56 days (Q1 – Q3: 52 – 63), respectively. All the obtained data are summarised in **Table 1**. **Supplementary File 2** contains the results of Kruskal-Wallis and Mann-Whitney *post-hoc* tests for the studied phenological traits, showing significant differences at the population level and between the assumed population groups. The distribution of these phenological traits is visually represented using box plots in **Figure 4**.

A correlation analysis revealed several significant associations among the studied traits (**Table 2**). A weak positive correlation was observed between FPD and VPD, while a strong positive correlation was found between VPD and BOF.

**Table 2:** Spearman’s correlation coefficients and *p*-values for the four *C. litardierei* phenological traits: FPD, VPD, BOF, and BOS.

Trait 1	Trait 2	Spearman’s ρ	*p*-value
FPD	VPD	0.025	0.725
FPD	BOF	-0.241	0.0005
FPD	BOS	-0.069	0.324
VPD	BOF	0.430	1.33e^-10^
VPD	BOS	-0.948	< 2.2e^-16^
BOF	BOS	-0.241	0.0005

BOF, Beginning of Flowering; BOS, Beginning of Sprouting; FPD, Flowering Period Duration; VPD, Vegetation Period Duration.

### Sequencing, genomic data processing, and population genetic structure

The sequencing process generated a total of 1,284,680,304 reads. After filtering the raw sequences and mapping them to the reference genome, 1,278,409,966 reads were retained. SNP identification and filtration were performed using the Stacks software, resulting in the detection of 24,660 SNPs. Following the application of the BCFtools MAF filter with a 1% threshold, 23,315 SNPs were kept for subsequent analysis.

The cluster analysis based on the Bayesian model implemented in the ParallelStructure software revealed that the most likely number of genetic clusters was two (**Supplementary File 3**). One cluster corresponded to the group of populations from the dolomite bedrock habitat, while the remaining populations formed the other cluster (**Supplementary File 4**). Such structuring reflects the environmental preferences of the studied populations only to some extent, as populations from seashore and meadow habitats remained grouped without any differentiation among them.

### Genome-wide association analyses

**Figure 5:**
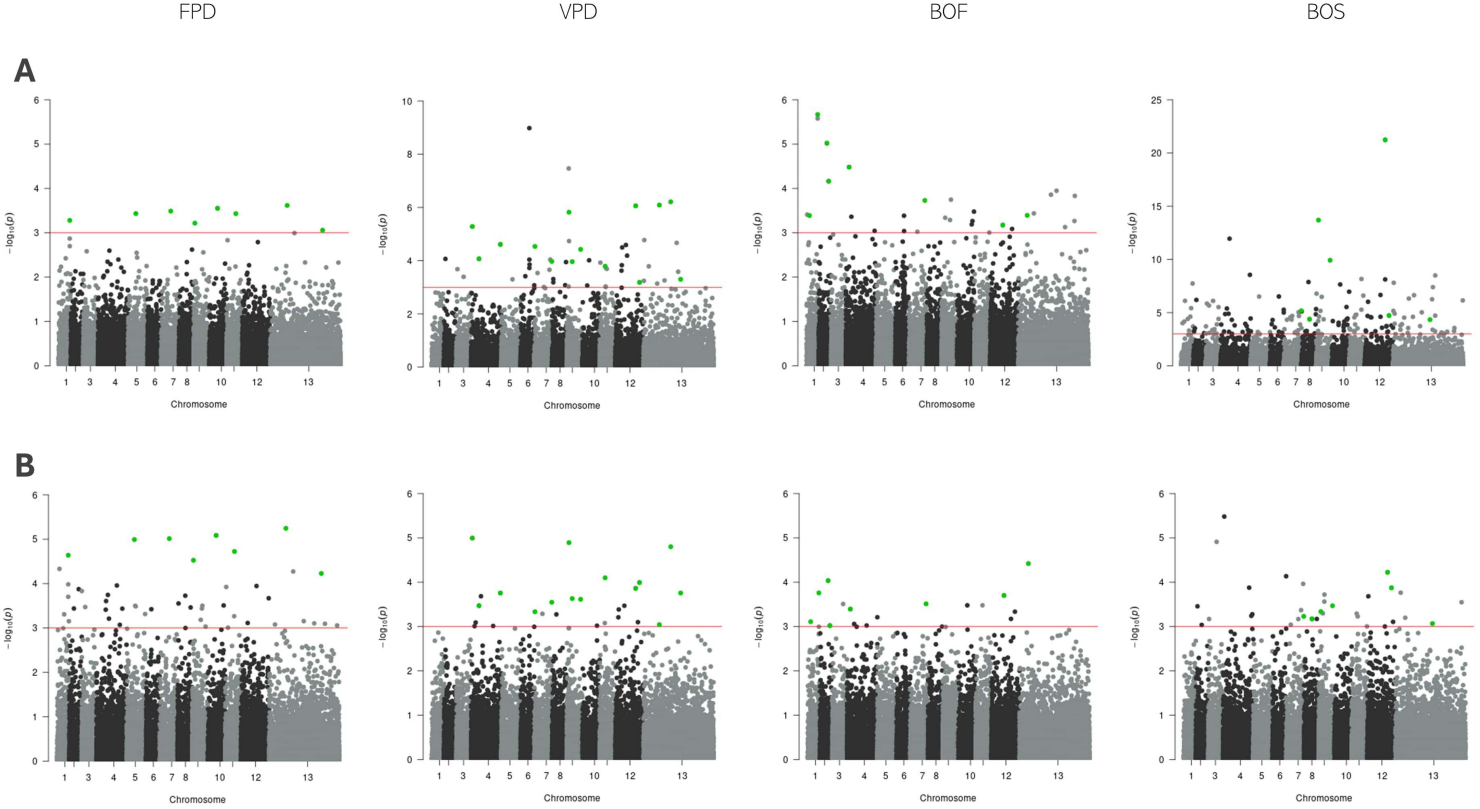
Manhattan plots of single-SNP association mapping of FPD, VPD, BOF, and BOS traits. Single-SNP analysis was conducted using **(A)** GMMAT (top row) and **(B)** GEMMA (bottom row) for each trait, where the x-axis represents the chromosomal positions of SNPs and the y-axis shows the −log10 (*p*-values) from the LMM analysis. The red horizontal line denotes the genome-wide significance threshold (*p* = 1 × 10^−^³). Each point on the Manhattan plot corresponds to a SNP, with stronger associations appearing higher due to lower *p*-values. Green dots indicate SNPs identified in both analyses.

The analysis of the FPD trait using LMM identified 48 significant SNPs, while GLMM detected 8. An overlap of these results revealed 8 SNPs that were significant across both methods. Further validation using BSLMM confirmed 3 of these SNPs as significant, with one located on each of chromosomes 10, 7, and 11. For the VPD trait, LMM identified 26 significant SNPs, while GLMM detected 54. Fourteen SNPs were found to overlap between the two methods. Subsequent analysis with BSLMM confirmed 2 of these SNPs as significant, located on chromosomes 4 and 12. In the case of the BOF trait, LMM and GLMM identified 17 and 29 SNPs, respectively, with 8 overlapping SNPs. BSLMM analysis confirmed 1 significant SNP located on chromosome 2. For the BOS trait, LMM identified 34 significant SNPs, while GLMM detected 162. Seven SNPs overlapped between the two methods, and BSLMM analysis confirmed 1 significant SNP on chromosome 12. All SNPs passing the genome-wide significance threshold (1 × 10^−3^) in both LMM and GLMM single-SNP LMM analysis are listed in **Table 3**. The results from the single-SNP association analysis conducted in GMMAT and GEMMA are presented together in Manhattan plots in **Figure 5**.

**Table 3:** SNPs passing the genome-wide significance threshold (1 × 10^−^³) in both GMMAT and GEMMA single-SNP LMM analyses for *Chouardia litardierei* traits: FPD, VPD, BOF, and BOS.

Trait	SNP	Chr	Position	Effect Allele	Referent Allele	MAF	Single-SNP LMM Analysis *β* (*p*-value) in GMMAT	Single-SNP LMM Analysis *β* (*p*-value) in GEMMA
FPD	275195_16	13	197688818	C	T	0.14	0.12 (2.41 × 10^-4^)	-0.52 (5.68 × 10^-6^)
FPD	131957_13	10	97222552	T	G	0.02	0.30 (2.79 × 10^-4^)	-1.13 (8.22 × 10^-6^)
FPD	750129_37	7	113120650	G	A	0.06	0.18 (3.24 × 10^-4^)	-0.76 (9.69 × 10^-6^)
FPD	688820_29	5	89511430	T	C	0.03	0.33 (3.68 × 10^-4^)	-1.39 (1.02 × 10^-5^)
FPD	134834_42	11	108997955	G	C	0.02	0.38 (3.69 × 10^-4^)	-1.49 (1.89 × 10^-5^)
FPD	445498_105	1	133595095	T	G	0.07	0.16 (5.24 × 10^-4^)	-0.66 (2.29 × 10^-5^)
FPD	53032_22	9	14725086	A	G	0.06	0.16 (6.05 × 10^-4^)	-0.68 (2.98 × 10^-5^)
FPD	380447_37	13	615321041	T	A	0.06	0.18 (8.73 × 10^-4^)	-0.77 (5.91 × 10^-5^)
VPD	565532_39	4	14626431	C	A	0.13	0.10 (5.23 × 10^-6^)	-0.46 (1.01 × 10^-5^)
VPD	65720_38	9	26233589	A	G	0.03	-0.14 (1.52 × 10^-6^)	0.60 (1.28 × 10^−5^)
VPD	305761_25	13	320423026	T	G	0.13	-0.08 (6.14 × 10^-7^)	0.33 (1.58 × 10^−5^)
VPD	167223_27	11	64125165	T	G	0.09	0.11 (1.62 × 10^-4^)	-0.46 (7.95 × 10^−5^)
VPD	221833_73	12	284678317	C	G	0.35	0.05 (6.52 × 10^-4^)	-0.22 (1.02 × 10^−4^)
VPD	210123_39	12	239066297	T	G	0.03	-0.13 (8.71 × 10^-7^)	0.51 (1.37 × 10^−4^)
VPD	618657_20	4	345766799	A	G	0.01	-0.23 (2.46 × 10^-5^)	0.93 (1.75 × 10^−4^)
VPD	334377_114	13	437172692	C	A	0.01	-0.14 (5.03 × 10^-4^)	0.75 (1.75 × 10^−4^)
VPD	76416_37	9	64503815	A	G	0.06	-0.08 (1.09 × 10^-4^)	0.35 (2.34 × 10^−4^)
VPD	57078_21	9	163441584	A	C	0.28	-0.06 (3.76 × 10^-5^)	0.23 (2.43 × 10^−4^)
VPD	774777_66	7	206933711	C	T	0.03	-0.14 (1.07 × 10^-4^)	0.56 (2.83 × 10^−4^)
VPD	635043_17	4	93373391	T	C	0.08	-0.09 (8.57× 10^-5^)	0.37 (3.38 × 10^−4^)
VPD	790473_18	7	8052404	A	T	0.02	-0.20 (2.39 × 10^-5^)	0.81 (4.63 × 10^−4^)
VPD	272420_33	13	186297710	T	G	0.08	-0.10 (8.13 × 10^-7^)	0.33 (9.05 × 10^−4^)
BOF	445520_34	1	133744238	A	G	0.23	-0.19 (2.16 × 10^-6^)	0.41 (1.74 × 10^−4^)
BOF	504422_54	2	95535920	T	G	0.34	-0.18 (9.52 × 10^-6^)	0.49 (9.17 × 10^−5^)
BOF	623094_18	4	39016369	A	G	0.43	-0.14 (3.29 × 10^-5^)	0.30 (4.02 × 10^−4^)
BOF	477240_15	2	115724781	A	G	0.25	-0.15 (6.87 × 10^-5^)	0.34 (9.49 × 10^−4^)
BOF	768498_16	7	186900792	G	C	0.02	-0.33 (1.86 × 10^-4^)	0.89 (3.07 × 10^−4^)
BOF	252813_22	13	104630774	C	G	0.03	0.60 (4.04 × 10^-4^)	-1.26 (3.79 × 10^−5^)
BOF	455458_35	1	37036194	G	T	0.36	-0.28 (4.08 × 10^-4^)	0.59 (7.76 × 10^−4^)
BOF	186978_19	12	148693882	A	C	0.03	-0.30 (6.69 × 10^-4^)	0.81 (1.99 × 10^−4^)
BOS	210123_39	12	239066297	T	G	0.03	0.95 (5.88 × 10^-22^)	-0.64 (5.95 × 10^-5^)
BOS	65720_38	9	26233589	A	G	0.03	0.71 (2.04 × 10^−14^)	-0.64 (5.95 × 10^−5^)
BOS	57078_21	9	163441584	A	C	0.28	0.20 (1.19 × 10^−10^)	-0.61 (4.60 × 10^−4^)
BOS	774777_66	7	206933711	C	T	0.03	0.36 (6.64 ×10^−6^)	-0.27 (3.39 × 10^−4^)
BOS	221833_73	12	284678317	C	G	0.35	-0.12 (1.85 × 10^-5^)	-0.66 (5.88 × 10^−4^)
BOS	38821_38	8	94422071	G	A	0.27	0.12 (4.12 × 10^-5^)	0.27 (1.34 × 10^−4^)
BOS	333922_26	13	435879200	T	G	0.43	0.12 (4.58 × 10^-5^)	-0.24 (6.68 × 10^−4^)

Statistical analyses were performed with GEMMA and GMMAT LMM. *p*-values < 1 × 10^−3^ are considered genome-wide significant. BOS, Beginning of Sprouting; BOF, Beginning of Flowering; Chr, Chromosome; FPD, Flowering Period Duration; LMM, Linear Mixed Model; MAF, Minor Allele Frequency; SNP, Single Nucleotide Polymorphism; VPD, Vegetation Period Duration.

In the Bayesian association analysis, two SNPs were identified as having a major sparse effect on the FPD trait. These SNPs were estimated to have a sparse effect in at least 10% of the BSLMM chain iterations (posterior inclusion probability, PIP ≥ 0.099). Additionally, both SNPs showed a sparse effect in over 16% of the iterations (PIP ≥ 0.165), further highlighting their significance. In contrast, for the VPD trait, 75 SNPs displayed a major sparse effect in ≥10% of BSLMM chain iterations (PIP ≥ 0.095). In addition, the top four SNPs displayed a major sparse effect in more than 44% of iterations (PIP ≥ 0.447). Concerning the BOF trait, three SNPs were identified with a major sparse effect in ≥10% of iterations (PIP ≥ 0.098) and the top SNP had a major sparse effect in over 17% of iterations (PIP ≥ 0.172). Similarly, for the BOS trait, 26 SNPs exhibited a sparse effect in ≥10% of BSLMM chain iterations (PIP ≥ 0.095), with the top two SNPs showing a strong effect in over 82% of iterations (PIP > 0.829). The data outlined above is reported in **Supplementary File 5**.

A total of 7 SNPs passed the genome-wide significance threshold (1 × 10^−^³) in the single-SNP LMM analyses and the posterior inclusion probability threshold (PIP ≥ 10%) in the Bayesian multi-SNP BSLMM analysis and are listed in **Table 4**. Manhattan plots from the BSLMM analysis are provided in **Supplementary File 6**.

**Table 4:** SNPs passing the genome-wide significance threshold (1 × 10^−^³) in the single-SNP LMM analyses and the posterior inclusion probability treshold (PIP ≥ 10%) in the Bayesian multi-SNP BSLMM analysis.

Trait	SNP	Chr	Position	Effect Allele	Referent Allele	MAF	Single-SNP LMM Analysis *β* (*p*-value) in GMMAT	Single-SNP LMM Analysis *β* (*p*-value) in GEMMA	Multi-SNP BSLMM Analysis *β* (PIP)
FPD	131957_13	10	97222552	T	G	0.02	0.30 (2.79 × 10^-4^)	-1.13 (8.22 × 10^-6^)	-0.70 (0.17)
FPD	750129_37	7	113120650	G	A	0.06	0.18 (3.24 × 10^-4^)	-0.76 (9.69 × 10^-6^)	-0.48 (0.17)
FPD	134834_42	11	108997955	G	C	0.02	0.38 (3.69 × 10^-4^)	-1.49 (1.89 × 10^-5^)	-0.70 (0.06)
VPD	565532_39	4	14626431	C	A	0.13	0.10 (5.23 × 10^-6^)	-0.46 (1.01 × 10^-5^)	-0.33 (0.91)
VPD	210123_39	12	239066297	T	G	0.03	-0.13 (8.71 × 10^-7^)	0.51 (1.37 × 10^-4^)	0.33 (0.75)
BOF	504422_54	2	95535920	T	G	0.34	-0.18 (9.52 × 10^-6^)	0.49 (9.17 × 10^-5^)	0.32 (0.17)
BOS	210123_39	12	239066297	T	G	0.03	0.95 (5.88 × 10^-22^)	-0.64 (5.95 × 10^-5^)	-0.48 (0.83)

Statistical analyses were performed with GEMMA and GMMAT LMM and BSLMM. *p*-values< 1 × 10^−3^ are considered genome-wide significant. BOS, Beginning of Sprouting; BOF, Beginning of Flowering; BSLMM, Bayesian Sparse Linear Mixed Model; Chr, Chromosome; FPD, Flowering Period Duration; LMM, Linear Mixed Model; MAF, Minor Allele Frequency; PIP; Posterior Inclusion Probability; SNP, Single Nucleotide Polymorphism; VPD, Vegetation Period Duration. The table presents the single-SNP LMM *p*-values along with their corresponding posterior inclusion probabilities from the BSLMM analysis for *Chouardia litardierei* traits FPD, VPD, BOF, and BOS.

### SNP heritability estimation

The BSLMM analysis, performed using 23,315 SNPs, provided estimates of narrow-sense heritability (PVE) for the phenological traits studied, along with the proportion of genetic effect (PGE) and the count of variants with a major effect (n.gamma), as detailed in **Table 5**. The PVE estimate for the FPD revealed that 20.26% of the phenotypic variation was explained by all available genotypes, with 47.22% attributed to 60 SNPs exhibiting significant phenotypic effects. Similarly, the PVE estimate for the VPD indicated that 86.95% of the phenotypic variation was explained by all genotypes, with 65.72% attributed to 111 SNPs exhibiting notable phenotypic effects. Moreover, the BSLMM analysis revealed that 66.03% of the phenotypic variation in BOF was explained by all genotypes, with 25.86% of this variation accounted for by 47 SNPs with significant effects. The PVE estimate for the BOS revealed that 76.05% of the phenotypic variation was explained by all available genotypes, with 63.19% attributed to 52 SNPs exhibiting significant phenotypic effects. **Supplementary File 7** contains the means, medians, and 95% equal tail posterior probability intervals (95% ETPPIs) of the hyperparameters derived from the BSLMM.

**Table 5:** Genetic architectures of *Chouardia litardierei* phenological traits identified using a BSLMM.

Trait	PVE/%	PGE/%	n.gamma
FPD	20.26	47.22	60
VPD	86.95	65.72	111
BOF	66.03	25.86	47
BOS	76.05	63.19	52

BOF, Beginning of Flowering; BOS, Beginning of Sprouting; FPD, Flowering Period Duration; n.gamma, number of variants with major effect; PGE, Proportion of Variance Explained by major effect variants; PVE, Proportion of Variance Explained by genetic data; VPD, Vegetation Period Duration.

### Multivariate GWAS analysis

**Figure 6:**
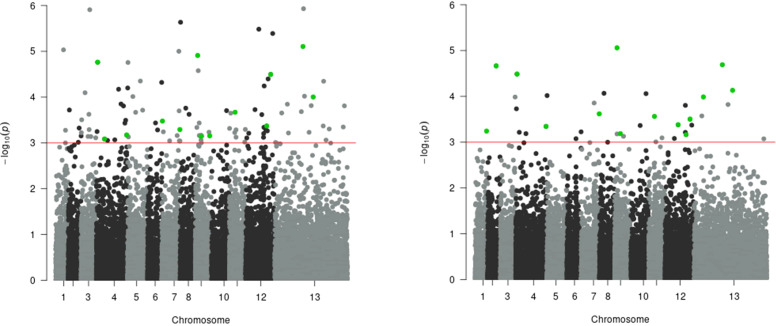
Manhattan plot of multivariate genome-wide association study (multi-GWAS) of **(A)** BOS and VPD (left) and **(B)** BOF and VPD traits (right). The red horizontal line indicates the genome-wide significance threshold (*p* = 1 × 10^-3^). Each dot on the Manhattan plot signifies a SNP. The strongest associations have the smallest *p*-values, so their negative logarithms will be the greatest, appearing higher on the plot. Green dots indicate SNPs identified as significant in the multivariate GWAS analysis as well as in both GEMMA and GMMAT univariate analyses for each of the two plots.

In the multivariate GWAS analysis, 113 SNPs surpassed the genome-wide significance threshold (*p* = 1 × 10^-3^) for the model with BOS and VPD traits as dependent variables (**Supplementary File 8**). This indicates shared genetic factors influencing these phenological traits across multivariate and univariate analyses. Five SNPs were significant in both LMM and GLMM univariate analyses for the BOS trait, and these same five were also significant for the VPD trait, along with an additional eight SNPs that were significant only for VPD, bringing the total to 13 (**Table 6**). In the multivariate GWAS analysis for the model with VPD and BOF traits as dependent variables, 36 SNPs exceeded the same threshold (**Supplementary File 9**). Among these, 10 SNPs were significant in LMM and GLMM univariate analyses for the VPD trait, while 4 showed significance for the BOF trait (**Table 6**). The multivariate GWAS findings for BOS and VPD, and BOF and VPD are plotted in Manhattan plots in **Figure 6**. The frequencies of effect alleles across populations for the significant SNPs (shown in **Tables 4**, **6**) are depicted in a plot provided in **Supplementary File 10**.

**Table 6:** SNPs passing genome-wide significance threshold (1 × 10^−3^) in the multivariate GWAS mvLMM analysis of *Chouardia litardierei* phenological traits BOS and VPD, and BOF and VPD.

Trait	SNP	Chr	Position	Effect Allele	Ref. Allele	MAF	Beta1 (VPD)	Beta2 (BOS)	mvLMM in GEMMA (*p*-value)
BOS +VPD	305761_25	13	320423026	T	G	0.13	0.35	-0.37	7.85 × 10^−6^
BOS +VPD	65720_38	9	26233589	A	G	0.03	0.61	-0.59	1.25 × 10^−5^
BOS +VPD	565532_39	4	14626431	C	A	0.13	-0.45	0.55	1.73 × 10^−5^
BOS +VPD	221833_73	12	284678317	C	G	0.35	-0.23	0.30	3.20 × 10^−5^
BOS +VPD	334377_114	13	437172692	C	A	0.01	0.75	-0.62	9.93 × 10^−5^
BOS +VPD	167223_27	11	64125165	T	G	0.09	-0.46	0.49	2.14 × 10^−4^
BOS +VPD	790473_18	7	8052404	A	T	0.02	0.85	-1.13	3.34 × 10^−4^
BOS +VPD	210123_39	12	239066297	T	G	0.03	0.51	-0.58	4.28 × 10^−4^
BOS +VPD	774777_66	7	206933711	C	T	0.03	0.57	-0.72	5.10 × 10^−4^
BOS +VPD	618657_20	4	345766799	A	G	0.01	0.93	-1.05	6.71 × 10^−4^
BOS +VPD	57078_21	9	163441584	A	C	0.28	0.22	-0.21	6.98 × 10^−4^
BOS +VPD	76416_37	9	64503815	A	G	0.06	0.35	-0.38	7.19 × 10^−4^
BOS +VPD	635043_17	4	93373391	T	C	0.08	0.37	-0.44	8.20 × 10^−4^
							Beta1 (VPD)	Beta2 (BOF)	
BOF +VPD	65720_38	9	26233589	A	G	0.03	0.59	0.25	8.71 × 10^−6^
BOF +VPD	305761_25	13	320423026	T	G	0.13	0.34	0.01	2.04 × 10^−5^
BOF +VPD	565532_39	4	14626431	C	A	0.13	-0.45	0.15	3.26 × 10^−5^
BOF +VPD	334377_114	13	437172692	C	A	0.10	0.73	0.49	7.39 × 10^−5^
BOF +VPD	774777_66	7	206933711	C	T	0.03	0.61	-0.27	2.41 × 10^−4^
BOF +VPD	167223_27	11	64125165	T	G	0.09	-0.46	0.16	2.74 × 10^−4^
BOF +VPD	221833_73	12	284678317	C	G	0.35	-0.22	0.11	3.14 × 10^−4^
BOF +VPD	618657_20	4	345766799	A	G	0.01	0.95	-0.28	4.52 × 10^−4^
BOF +VPD	76416_37	9	64503815	A	G	0.06	0.35	0.06	6.51 × 10^−4^
BOF +VPD	210123_39	12	239066297	T	G	0.03	0.50	-0.01	6.84 × 10^−4^
BOF +VPD	504422_54	2	95535920	T	G	0.34	0.23	0.49	2.16 × 10^−5^
BOF +VPD	252813_22	13	104630774	C	G	0.03	0.10	-1.27	1.03 × 10^−4^
BOF +VPD	186978_19	12	148693882	A	C	0.03	-0.22	0.82	4.17 × 10^−4^
BOF +VPD	445520_34	1	133744238	A	G	0.23	0.06	0.39	5.72 × 10^−4^

Statistical analyses were performed with GEMMA mvLMM. *p*-values< 1 × 10^−3^ are considered genome-wide significant. BOF, Beginning of Flowering; BOS, Beginning of Sprouting; Chr, Chromosome; FPD, MAF, Minor Allele Frequency; mvLMM, multivariate Linear Mixed Model; SNP, Single Nucleotide Polymorphism; VPD, Vegetation Period Duration. Listed SNPs were found to be significant in both GEMMA and GMMAT univariate analyses.

### GWAS candidate genes identification

The eggNOG tool provided detailed data clarifying the connection between individual SNPs/sequences and specific protein families (PFAM). To identify candidate genes potentially influencing phenological traits, we conducted eggNOG analysis on 7 SNPs that passed the genome-wide significance threshold (1 × 10^−^³) in both the single-SNP LMM and multi-SNP BSLMM analyses of *C. litardierei* traits, including FPD, VPD, BOF, and BOS. This analysis identified 59 queries corresponding to sequences matched to the eggNOG database for functional annotation (**Supplementary File 11**). Using eggNOG, we further analyzed 13 SNPs that met the same significance threshold in the multivariate GWAS analysis of BOS and VPD, uncovering 114 additional queries (**Supplementary File 12**). Similarly, 14 SNPs passed the same significance threshold in the multivariate GWAS analysis of VPD and BOF, resulting in 173 additional queries (**Supplementary File 13**). The eggNOG analysis connected sequences to protein families, which we further explored through manual inspection and a literature review to identify specific genes and PFAM domains related to the traits being studied. Some domains were common to both the univariate and multivariate GWAS results, resulting in overlaps. The most significant findings, along with their biological functions and relevant references, are summarized in **Table 7**.

**Table 7:** List of candidate genes for regions of strong association with FPD, VPD, BOF and BOS identified by the eggNOG-mapper v2 database.

Query	Method	*e*-value	Chr	EGGNog PFAM	Candidate Genes	Species	Relevant biological functions	References
H 9:113095650-113145650_6	GWAS	4.55e^-212^	7	Chromo domain	LHP1	*Arabidopsis* *thaliana*	Chromatin regulation and flowering time control	Gaudin et al. (2001), Adrian et al. (2010)
H 9:206908711-206958711_54	mGWAS1	6.8e^-111^	13					
H 2:95510920-95560920_36	mGWAS2	3.82e^-132^	2					
H 9:113095650-113145650_4	GWAS	3.1e^-163^	7	Histidine phosphatase protein family	Hd3a, ZCN8	*Oryza sativa*, *Zea mays*	Hormone signalling, development, stress response, and flowering	Cho et al. (2022)
H 9:113095650-113145650_44	GWAS	2.31e^-42^	7	Aspartic Proteases (APs)	PvAP1	*Phaseolus* *vulgaris*	Drought stress adaptation and osmotic resistance	Contour-Ansel et al. (2010)
H 4:14601431-14651431_21	GWAS	2.19e^-80^	4	CCHC-type zinc finger proteins (CCHC-ZFPs)	AtCSP4	*Arabidopsis* *thaliana*	Growth, development, and stress responses	Yang and Karlson (2011)
H 15:284653317-284703317_45	mGWAS1	1.77e^-306^	12					
H 15:284653317-284703317_50	mGWAS2	1.77e^-306^	12					
H 4:14601431-14651431_5	GWAS	1.14e^-23^	4	Pentatricopeptide repeat (PPR) proteins	AT1G15480	*Arabidopsis* *thaliana*	Flowering time regulation	Emami and Kempken (2019)
H 4:14601431-14651431_4	mGWAS2	9.61e^-26^	4					
H 14:108972955-109022955_19	GWAS	2.14e^-100^	11	Phytochrome-interacting Factor 1 (PIF1)	PIF1	*Arabidopsis* *thaliana*	Sprouting control, growth, stress adaptation, and photosynthesis regulation	Yadav (2024), Soy et al. (2014), Li et al. (2024), Chen et al. (2013)
H 14:108972955-109022955_22	mGWAS1	1.00e^-308^	11					
H 16:320398026-320448026_57	mGWAS1	3.11e^-22^	13	MATE domain	OsMATE2, OsMATE4, OsMATE42, OsMATE46	*Oryza sativa*	Early salt stress response and drought stress resistance	Du et al. (2021)
H 16:320398026-320448026_58	mGWAS2	3.11e^-22^	13					
H 15:284653317-284703317_6	mGWAS1	1.08e^-12^	12	Protein kinase domain	SOS2/CIPK24	*Arabidopsis* *thaliana*	Salt stress responses and hormonal signaling	Chen et al. (2023)
H 4:345741799-345791799_9	mGWAS2	2.09e^-24^	4					
H 1:133719238-133769238_38	mGWAS2	1.33e^-36^	1	MLO protein family	OsMLO1-4, OsMLO9, OsMLO11	*Oryza sativa*	Heat and/or cold stress response	Nguyen et al. (2016)
H 15:148668882-148718882_58	mGWAS2	5.59e^-190^	12	C2 domain	QUIRKY, STRUBBELIG	*Arabidopsis* *thaliana*	Promotes intercellular communication and tissue morphogenesis	Vaddepalli et al. (2014)

BOF, Beginning of Flowering; Chr, chromosome; BOS, Beginning of Sprouting; flowering period duration; GWAS, genome-wide association study; H, HiC scaffold; mGWAS1, multivariate Genome-Wide Association Study of BOS and VPD; mGWAS2, multivariate Genome-Wide Association Study of BOF and VPD; PFAM, protein family; VPD, Vegetation Period Duration. (*e*-value< 1 × 10^−2^) in *Chouardia litardierei* based on the 7 recognized SNPs passing genome-wide significance threshold (1 × 10^−3^) in the single-SNP LMM and multi-SNP BSLMM analysis as well as 13 SNPs passing the same threshold in the multivariate GWAS mvLMM analysis of BOS and VPD (mGWAS1), and 14 SNPs in BOF and VPD (mGWAS2). The names of the identified candidate genes associated with the SNPs, PFAMs, their relevant biological functions, and corresponding references are provided.

## Discussion

This study aimed to advance our understanding of the genetic foundations of phenological adaptive traits in *C. litardierei*’s populations occupying contrasting habitats and shaped by distinct ecological pressures. To minimize the effects of phenotypic plasticity and identify heritable local adaptation traits as accurately as possible, individuals from divergent environments were grown under uniform conditions (Liu and El-Kassaby, 2019; Schwinning et al., 2022). This approach allowed the separation of genetic influences from environmental effects, revealing the heritable components driving local adaptation, where populations evolve toward optimal phenotypic and genetic configurations in response to local selective pressures (Montejo-Kovacevich et al., 2021).

Basic statistical analyses on the common garden experiment data were first performed to characterize the variations within the tested phenological traits and their potential importance for the local adaptation of studied populations in their natural habitats. Except for the duration of flowering (FPD), substantial variations in tested traits among the studied populations were revealed, highlighting their importance for adaptation to contrasting environmental pressures. However, although significant differences in phenological traits among studied groups and individual populations were present, there were many exceptions in the general pattern. For instance, although the dolomite-habitat population group began with flowering (BOF) before the remaining two groups, the Pag population from the seashore habitat was an exception, as it overlaped with all the dolomite-habitat populations. At the same time, the Pag population came into flowering significantly earlier than the Vrana Lake population, which is found in the same habitat and is even geographically closely positioned to the Pag population. Similarly, VPD was significantly shorter in the dolomite-habitat group of populations in contrast to other groups; however, the Budoške Bare population from karst poljes’ meadow habitat joined this group due to having VPD also significantly shorter than any of the remaining populations from this and the seashore habitat. Such a result supports the earlier assumption that although groups of *C. litardierei* population thrive in highly contrasting habitats, their differentiation into well-differentiated ecotypes remains poorly supported. This was also partially confirmed by the obtained population genetic results (**Supplementary Files 3**, **4**). Here, only the group of populations from the dolomite habitat was substantially differentiated and formed a well-defined genetic cluster, while all the remaining populations remained clustered together, without signs of differentiation between the seashore and meadow-habitat groups. Since the ecotypes are defined as groups of populations whose differentiation is supported both genetically and phenotypically (Lowry, 2012), the studied groups do not meet these criteria. Nonetheless, some trends can be observed in the obtained results that point to certain conclusions. The dolomite-habitat population group sprouted later (BOS) and flowered earlier (BOF) but had a shorter vegetation period (VPD) than the remaining two groups. Such a shift in phenophases is likely to be significantly influenced by habitat properties. To understand how this specific habitat may affect this phenomenon, two aspects must be considered: the dolomite substrate properties and the influence of the local climate dynamics on the vegetation season. These southernmost populations of *C. litardierei* are usually found on bare dolomite bedrock or less frequently in dry, exposed mountainous grassland habitats developed on very shallow rendzina soils (**Figure 1**). Due to reduced water and nutrient capacity, accompanied by high levels of thermal conductivity and thermal capacity of the dolomite substrate (Thomas et al., 1973; Waples and Waples, 2004; Mota et al., 2021), these drought-prone habitats are known to induce heat stress in adjacent organisms and thus present a hostile environment for plant species (Mota et al., 2021). In addition, due to very sparse vegetation cover in such habitats, the substrate temperature can be expected to reach far greater values when compared to habitats covered with canopies or meadows (Oliver et al., 1987), thus further worsening already inhospitable conditions. Regarding the influence of regional climatic patterns on local vegetation, two peaks of ecosystem productivity have been observed in Mediterranean climate conditions across southern Europe – the larger one during spring and the less pronounced one during autumn. Such a modality has developed because of ecological constraints imposed by low winter temperatures on one side and summer droughts on another (Spano et al., 2013; Camarero et al., 2021), thus leaving relatively short time frames in spring and autumn suitable for development and reproduction. Consequently, it seems plausible that populations experiencing such climatic patterns, in combination with drought- and heat-stress-prone habitats, have developed short development- and reproduction-related phenophases. At the same time, the remaining *C. litardierei* populations inhabiting deep, moisture-retaining soils protected by dense vegetation layer which additionally reduces the increase of substrate temperature (Oliver et al., 1987), experience a less limited time frame for closing the sexual reproduction cycle. This is reflected in significant shifts in related phenophases toward later sprouting and the beginning of flowering, as well as a more extended vegetation period.

By emphasizing their heritable nature, the high PVE values observed in our study were suggested to indicate the great evolutionary importance of detected candidate loci in shaping the phenological adaptation of populations to local climatic conditions. The highest PVE value (86.95%) was exhibited by the trait VPD, suggesting that the length of the growing season in this species is predominantly determined by genetic factors. The high genetic variance observed in VPD could be reflective of adaptive mechanisms that allow *C. litardierei* to optimize its growth and reproductive success in response to environmental cues, such as climate and soil conditions, with strong natural selection acting on traits critical for survival in fluctuating environments. While a PVE for flowering time exceeding 95% has been reported for *Arabidopsis* from Cape Verde and Morocco (Neto and Hancock, 2023), highlighting the predominant genetic influence, the PVE for *C. litardierei* flowering period duration (FPD) was found to be 20.26%, indicating a more significant role of environmental or non-genetic factors. PVE values of 66.03% and 76.05% were exhibited by the BOF and BOS traits, respectively, indicating that genetic elements were exerting a greater influence than local environmental factors in shaping these traits. This was reinforced by the PGE values, with the highest PGE (65.72%) being observed in VPD, driven by a few major variants. In contrast, lower PGE values (25.86% and 63.19%) were found for BOF and BOS, respectively, reflecting the influence of numerous small-effect variants and a greater environmental impact. Overall, these heritability estimates and genetic findings provided evidence of the significant role played by genetic factors in shaping phenological traits in *C. litardierei*, emphasizing the complex interaction between genetics and environment and offering a strong foundation for future genetic, evolutionary, and adaptation studies.

In this study, multiple loci linked to phenological traits in *C. litardierei* were identified through univariate and multivariate GWAS approaches. The relatively low overlap of significant SNPs detected across the different GWAS models likely reflects inherent differences in their statistical assumptions and approaches to modelling genetic effects. While both frequentist methods (GLMM and LMM) applied a consistent significance threshold of < 1 × 10^−3^, the BSLMM relies on posterior inclusion probabilities, which are generally more conservative and not directly comparable to *p*-values. Importantly, each model is optimized for different data characteristics: LMM assumes normally distributed traits, whereas GLMM, using a Poisson distribution, is more appropriate for count-based traits with non-normal distributions. Applying trait-appropriate models increases the reliability and power of association detection, even if it results in a lower number of shared SNPs. Functional annotation of the genomic windows surrounding significant SNP loci revealed regions encoding key protein families involved in essential biological pathways related to phenological events. Among others, SNP loci were identified in regions encoding the chromo domain, which is crucial to plant chromatin-based gene regulation. In *Arabidopsis*, mutations in LHP1 (LIKE HETEROCHROMATIN PROTEIN 1), a gene encoding a chromo domain, have been shown to cause early flowering and reduced plant size (Gaudin et al., 2001). Overexpression of CONSTANS (CO), which activates FLOWERING LOCUS T (FT) in long-day conditions, has been found to alter chromatin at the FT locus by reducing LHP1 binding and increasing histone acetylation, suggesting LHP1 represses flowering through chromatin regulation (Adrian et al., 2010). SNP loci were also identified in regions encoding histidine phosphatase proteins, which are known to regulate plant development and stress responses, particularly through hormone signaling pathways like cytokinins that influence flowering and vegetative growth (Werner et al., 2001; Hai et al., 2020). For instance, it has been demonstrated that exogenous cytokinin application extends the vegetative phase in rice and maize by inhibiting the expression of florigen genes, such as Hd3a and ZCN8, thus delaying flowering time (Cho et al., 2022). Additionally, cytokinins have been found to interact with environmental signals like nutrient sensing (Argueso et al., 2009; Prasad, 2022), potentially aiding plant adaptation to nutrient-poor and drought-prone habitats, like those inhabited by the southern group of *C. litardierei* populations. Similarly, cytokinin-deficient mutants have been observed to exhibit delayed flowering on nutrient-poor substrates, underscoring cytokinin’s role in adaptation to nutrient-limited environments (Miyawaki et al., 2006). SNP loci within the genomic regions encoding aspartic proteases (APs) and CCHC-type zinc finger proteins (CCHC-ZFPs) were recognized as well. In drought-susceptible common bean cultivars, the PvAP1 gene exhibited significant upregulation under mild water stress, supporting the role of APs in drought responses (Contour-Ansel et al., 2010). CCHC-ZFPs are considered essential for growth and development, as demonstrated in *Arabidopsis*, where AtCSP4 has been identified as a key factor (Yang and Karlson, 2011). Additionally, SNP loci within the genomic region encoding pentatricopeptide repeat (PPR) proteins were identified. It has been reported that mutations in the *Arabidopsis* gene AT1G15480, encoding a P-class PPR protein, result in early flowering (Emami and Kempken, 2019). Furthermore, mutations were detected in genetic regions responsible for encoding phytochrome-interacting factor 1 (PIF1). In *Arabidopsis*, PIF1 has been found to play a major role in sprouting inhibition (Oh et al., 2004; Yadav, 2024), plant growth and development regulation (Soy et al., 2014), stress adaptation (Li et al., 2024), and regulation of photosynthesis initiation (Chen et al., 2013). In addition, SNP loci were identified within regions encoding the MATE domain, the protein kinase domain, and loci associated with the MLO protein family. Several MATE domain genes in *O. sativa* (OsMATE2, OsMATE4, OsMATE42, and OsMATE46) have been shown to regulate plant responses to abiotic stresses, such as salt and drought, through differential expression patterns (Du et al., 2021), while the protein kinase SOS2/CIPK24 has been recognized as a central regulator of salt stress response and hormonal signaling in *Arabidopsis* (Chen et al., 2023). Finally, the MLO protein family is considered crucial for temperature stress adaptation, as exemplified by several OsMLO proteins in *O. sativa* (Nguyen et al., 2016).

Here, we investigated the genetic background of phenological traits in *C. litardierei*, revealing significant associations between them and specific genetic variations across the genome. Our findings indicate that certain genomic regions may be instrumental in the adaptive responses of populations to contrasting environmental conditions. The genetic architecture of these phenological traits is complex, with multiple candidate loci contributing to phenotypic diversity across habitats. Using the ddRAD-seq approach and comprehensive GWAS analyses, we identified key candidate genes and multiple loci associated with phenological traits. However, the limited genome scan resolution of ddRAD-seq, particularly in large genomes like *C. litardierei* (3.7 Gb), leaves much genomic information unexplored. The relatively small sample size is a limitation of our study, particularly given that GWAS typically include larger cohorts to detect robust and reproducible associations. Nevertheless, our analysis revealed several biologically plausible signals, which, while requiring validation, provide a valuable foundation for future studies. These findings should be interpreted with caution, but they offer meaningful insights that can be further explored and confirmed in larger, independent populations. Functional annotation of the associated genomic regions revealed key protein families involved in vital biological pathways related to flowering time, vegetative growth, and stress adaptation. These protein families are crucial regulators of plant development, environmental responses, and abiotic stress adaptation. High narrow-sense heritability estimates indicated that genetic factors accounted for a significant portion of the phenotypic variance, with PVE ranging from 20.26% for flowering period duration (FPD) to 86.95% for vegetation period duration (VPD). This study underscores the complexity of the genetic architecture driving phenotypic diversity in plants, highlighting the critical role of genomic approaches in examining adaptive traits in non-model species exposed to diverse ecological pressures. Despite challenges in studying a wild, non-model species, this research advances our understanding of the genomic basis of adaptive divergence and ecological differentiation in *C. litardierei*. Expanding this research through a comprehensive Genome-Environment Association (GEA) study, incorporating more populations across the species’ distribution range, could provide deeper insights into the genomic drivers of local adaptation and phenological divergence.

## Data Availability

The datasets presented in this study can be found in online repositories. The names of the repository/repositories and accession number(s) can be found below: https://www.ncbi.nlm.nih.gov/, PRJNA1164649.

## Glossary

APs: Aspartic Proteases
BAM: Binary Alignment Map
BOF: Beginning of flowering
BOS: Beginning of sprouting
BSLMM: Bayesian Sparse Linear Mixed Model
CCHC-ZFPs: CCHC-type zinc finger proteins
Chr: Chromosome
ddRAD-seq: Double Digest Restriction Site-Associated DNA Sequencing
EGGNog: Evolutionary Genealogy of Genes: Non-supervised Orthologous Groups
FPD: Flowering period duration
GEA: Genome-Environment Association
GEMMA: Genome-wide Efficient Mixed Model Association
GMMAT: Generalized Mixed Model Association Tests
GWAS: Genome-Wide Association Study
LMM: Linear Mixed Model
MAF: Minor Allele Frequency
mGWAS: Multivariate genome-wide association study
PGE: Proportion of Genetic Effect
PPR proteins: Pentatricopeptide repeat proteins
PFAM: Protein Family
PIF1: Phytochrome-interacting Factor 1
PIP: Posterior Inclusion Probability
PVE: Proportion of Variance Explained
SNP: Single Nucleotide Polymorphism
VPD: Vegetation period duration
