# Supplementary material for: Uncovering the genomic basis of phenological traits in Chouardia litardierei (Asparagaceae) through a genome-wide association study (GWAS)
Source: Front Plant Sci. 2025 Apr 17;16:1571608. doi: 10.3389/fpls.2025.1571608 (PMC12070586; doi:10.3389/fpls.2025.1571608)
Supplement: Supplementary file 1 [file Table1.docx]

**Table 1.** The locations of sampled *Chouardia litardierei* populations and their corresponding habitat types.

| **Population/Location** | **Country** | **Latitude (N)** | **Longitude (E)** | **Habitat Type** |
| --- | --- | --- | --- | --- |
| Bjelopolje | Croatia | 44.693754° | 15.773682° | Meadow – karst poljes |
| Cetina (Paško polje) | Croatia | 43.940922° | 16.436367° | Meadow – karst poljes |
| Budoške Bare | Montenegro | 42.743747° | 18.926361° | Meadow – karst poljes |
| Pag (Kolansko blato) | Croatia | 44.514886° | 14.919922° | Seashore - grassland |
| Nin | Croatia | 44.249564° | 15.172015° | Seashore - grassland |
| Vrana Lake | Croatia | 43.937292° | 15.514689° | Seashore - grassland |
| Lovćen | Montenegro | 42.377169° | 18.843117° | Dolomite - bedrock |
| Skadar Lake | Montenegro | 42.326486° | 19.069464° | Dolomite - bedrock |
| Pandurica | Montenegro | 42.721628° | 18.962442° | Dolomite - bedrock |
