# Supplementary material for: Uncovering the genomic basis of phenological traits in Chouardia litardierei (Asparagaceae) through a genome-wide association study (GWAS)
Source: Front Plant Sci. 2025 Apr 17;16:1571608. doi: 10.3389/fpls.2025.1571608 (PMC12070586; doi:10.3389/fpls.2025.1571608)
Supplement: Supplementary file 2 [file Table2.docx]

| **Table 1.** Mann-Whitney post-hoc test for four phenological traits of *Chouardia litardierei*. | | | |
| --- | --- | --- | --- |
| **trait** | **continental-litorarl** | **continental-dolomitic** | **litoral-dolomitic** |
| **BOF** | 0.5425 | 1.48E-06 | 7.32E-09 |
| **BOS** | 1 | 4.13E-12 | 1.04E-18 |
| **FPD** | NA | NA | NA |
| **VPD** | 0.918 | 1.34E-15 | 3.09E-23 |
|  |  |  |  |
|  |  |  |  |
|  |  |  |  |
| **Table 2.** Kruskal-Wallis test for equal medians for four phenological traits of *Chouardia litardierei*. | | | |
| **trait** | **H (chi2)** | **Hc (tie corrected)** | ***p* (same)** |
| **BOF** | 41.69 | 42.4 | 6.22E-10 |
| **BOS** | 86.83 | 87.4 | 1.048E-16 |
| **FPD** | 0.1727 | 0.1746 | 0.9164 |
| **VPD** | 113.8 | 113.9 | 1.845E-25 |

**Figure 1.** Box plots showing the distribution of four phenological traits (BOF, BOS, FPD, and VPD) in *Chouardia* *litardierei* populations across different habitat types. Each box plot represents the median, interquartile range (IQR), and variability of trait values across different populations. Whiskers indicate data within 1.5 times the IQR, while dots represent outliers.


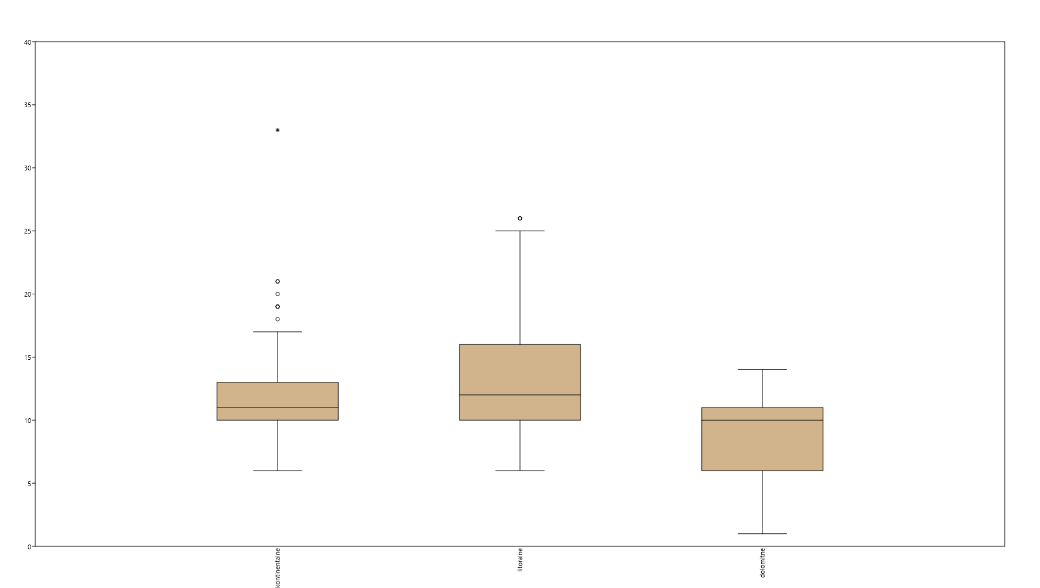


BOF


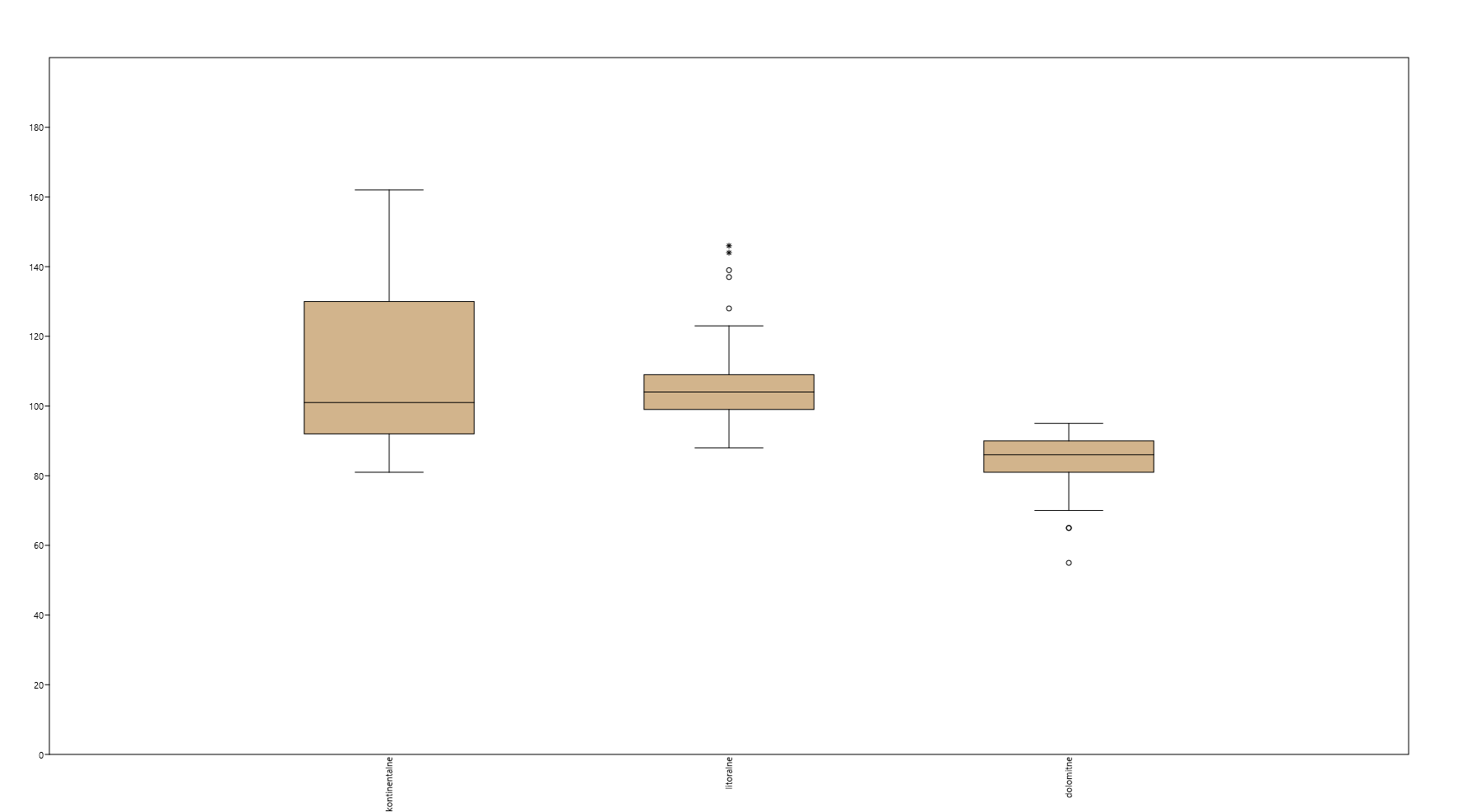


VPD


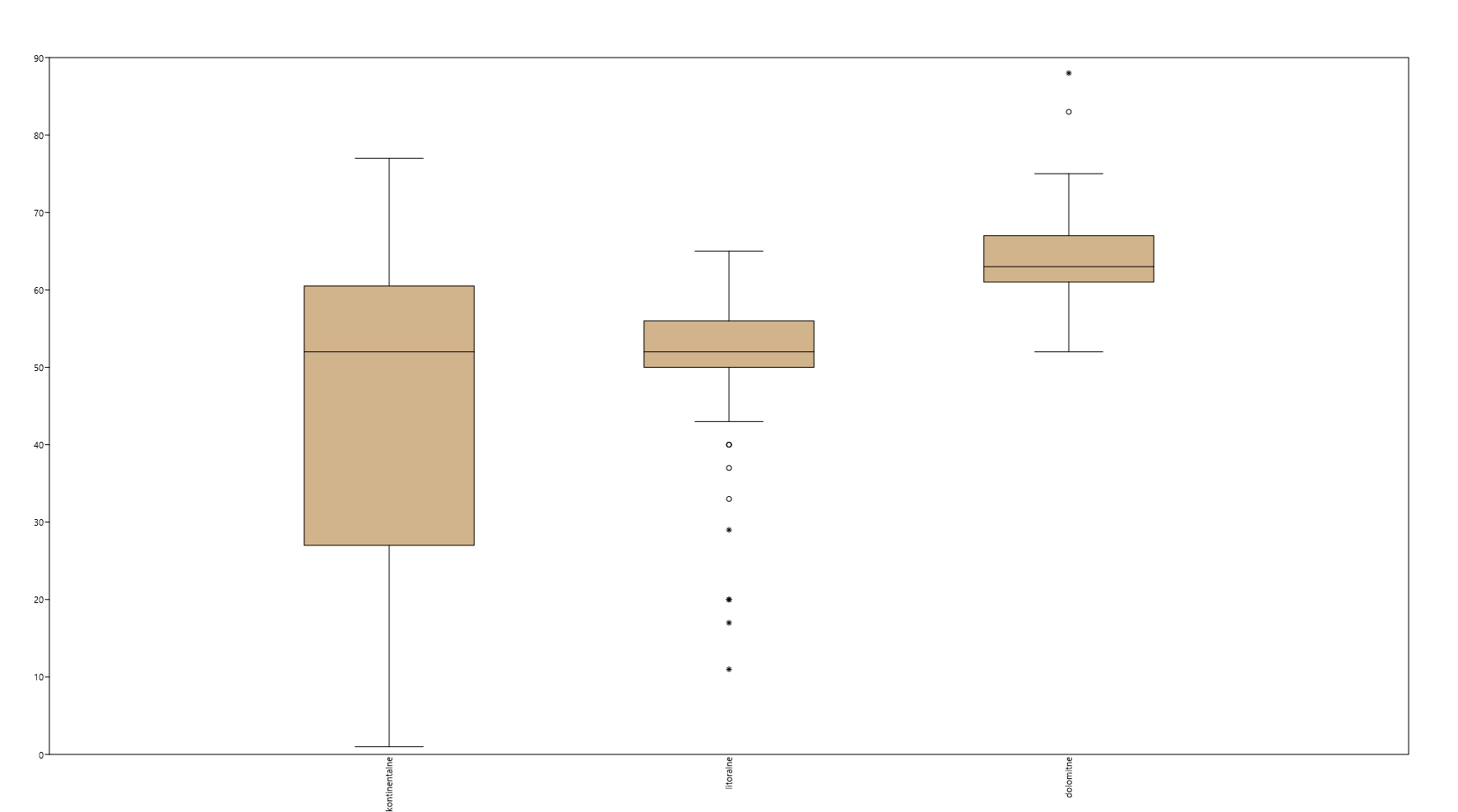

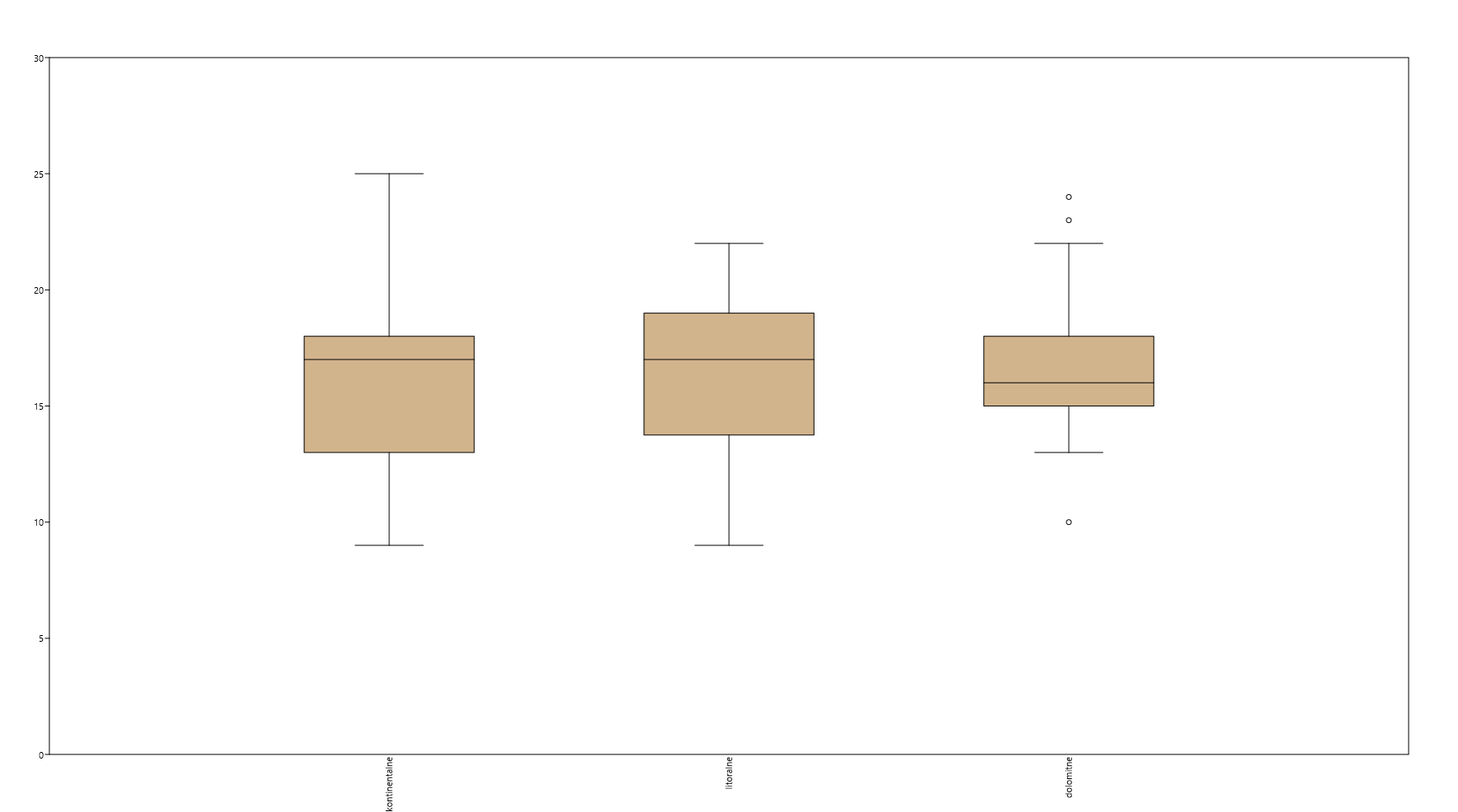


FPD

BOS

VPD

**Table 3.** Kruskal-Wallis test for equal medians for four phenological traits in nine *Chouardia litardierei* populations.

| **trait** | **H (chi2)** | **Hc (tie corrected)** | ***p* (same)** |
| --- | --- | --- | --- |
| **BOF** | 104 | 105.8 | 2.762E-19 |
| **BOS** | 141.5 | 142.5 | 7.243E-27 |
| **FPD** | 21.44 | 21.68 | 0.005543 |
| **VPD** | 168.2 | 168.3 | 2.961E-32 |

**Table 4.** Mann-Whitney post-hoc test for VPD trait in nine *Chouardia litardierei* populations.

|  | BJELOPOLJE | CETINA | BUDOŠKE BARE | VRANA LAKE | NIN | PAG | SKADAR LAKE | PANDURICA | LOVĆEN |
| --- | --- | --- | --- | --- | --- | --- | --- | --- | --- |
| BJELOPOLJE |  | 5.72E-05 | 2.79E-06 | 0.003915 | 2.23E-05 | 2.74E-06 | 1.15E-06 | 3.84E-06 | 1.16E-06 |
| CETINA | 5.72E-05 |  | 1.18E-05 | 0.03173 | 1 | 0.4125 | 2.31E-07 | 1.73E-06 | 1.82E-07 |
| BUDOŠKE BARE | 2.79E-06 | 1.18E-05 |  | 2.88E-07 | 2.07E-06 | 0.00124 | 0.1842 | 1 | 0.0003949 |
| VRANA LAKE | 0.003915 | 0.03173 | 2.88E-07 |  | 0.5151 | 3.51E-06 | 7.26E-08 | 3.62E-07 | 7.28E-08 |
| NIN | 2.23E-05 | 1 | 2.07E-06 | 0.5151 |  | 0.00987 | 7.83E-08 | 3.93E-07 | 7.85E-08 |
| PAG | 2.74E-06 | 0.4125 | 0.00124 | 3.51E-06 | 0.00987 |  | 1.16E-06 | 1.91E-05 | 5.55E-07 |
| SKADAR LAKE | 1.15E-06 | 2.31E-07 | 0.1842 | 7.26E-08 | 7.83E-08 | 1.16E-06 |  | 1 | 0.2871 |
| PANDURICA | 3.84E-06 | 1.73E-06 | 1 | 3.62E-07 | 3.93E-07 | 1.91E-05 | 1 |  | 0.002412 |
| LOVĆEN | 1.16E-06 | 1.82E-07 | 0.0003949 | 7.28E-08 | 7.85E-08 | 5.55E-07 | 0.2871 | 0.002412 |  |

**Table 5.** Mann-Whitney post-hoc test for FPD trait in nine *Chouardia litardierei* populations.

|  | BJELOPOLJE | CETINA | BUDOŠKE BARE | VRANA LAKE | NIN | PAG | SKADAR LAKE | PANDURICA | LOVĆEN |
| --- | --- | --- | --- | --- | --- | --- | --- | --- | --- |
| BJELOPOLJE |  | 0.052 | 0.02663 | 0.2822 | 0.2316 | 0.2235 | 1 | 0.05792 | 0.03659 |
| CETINA | 0.052 |  | 1 | 1 | 1 | 1 | 0.6153 | 1 | 1 |
| BUDOŠKE BARE | 0.02663 | 1 |  | 1 | 1 | 1 | 0.3502 | 1 | 1 |
| VRANA LAKE | 0.2822 | 1 | 1 |  | 1 | 1 | 1 | 1 | 1 |
| NIN | 0.2316 | 1 | 1 | 1 |  | 1 | 1 | 1 | 1 |
| PAG | 0.2235 | 1 | 1 | 1 | 1 |  | 1 | 1 | 1 |
| SKADAR LAKE | 1 | 0.6153 | 0.3502 | 1 | 1 | 1 |  | 1 | 1 |
| PANDURICA | 0.05792 | 1 | 1 | 1 | 1 | 1 | 1 |  | 1 |
| LOVĆEN | 0.03659 | 1 | 1 | 1 | 1 | 1 | 1 | 1 |  |

**Table 6.** Mann-Whitney post-hoc test for BOS trait in nine *Chouardia litardierei* populations.

|  | BJELOPOLJE | CETINA | BUDOŠKE BARE | VRANA LAKE | NIN | PAG | SKADAR LAKE | PANDURICA | LOVĆEN |
| --- | --- | --- | --- | --- | --- | --- | --- | --- | --- |
| BJELOPOLJE |  | 0.0014 | 1.37E-06 | 0.03928 | 0.00014 | 2.82E-06 | 2.21E-07 | 1.704E-07 | 2.2E-07 |
| CETINA | 0.0014 |  | 0.000529 | 1 | 1 | 0.4914 | 8.21E-06 | 0.0000121 | 1.1E-06 |
| BUDOŠKE BARE | 1.4E-06 | 0.00053 |  | 5E-06 | 0.0012 | 0.04781 | 1 | 1 | 0.2271 |
| VRANA LAKE | 0.03928 | 1 | 5E-06 |  | 1 | 0.000133 | 2.89E-07 | 2.661E-07 | 1.9E-07 |
| NIN | 0.00014 | 1 | 0.001198 | 1 |  | 1 | 2.71E-05 | 6.456E-05 | 2.2E-06 |
| PAG | 2.8E-06 | 0.4914 | 0.04781 | 0.00013 | 1 |  | 0.000483 | 0.0009476 | 1.4E-05 |
| SKADAR LAKE | 2.2E-07 | 8.2E-06 | 1 | 2.9E-07 | 2.7E-05 | 0.000483 |  | 1 | 0.2374 |
| PANDURICA | 1.7E-07 | 1.2E-05 | 1 | 2.7E-07 | 6.5E-05 | 0.000948 | 1 |  | 0.04234 |
| LOVĆEN | 2.2E-07 | 1.1E-06 | 0.2271 | 1.9E-07 | 2.2E-06 | 1.41E-05 | 0.2374 | 0.04234 |  |

**Table 7.** Mann-Whitney post-hoc test for BOF trait in nine *Chouardia litardierei* populations.

|  | BJELOPOLJE | CETINA | BUDOŠKE BARE | VRANA LAKE | NIN | PAG | SKADAR LAKE | PANDURICA | LOVĆEN |
| --- | --- | --- | --- | --- | --- | --- | --- | --- | --- |
| BJELOPOLJE |  | 0.004181 | 0.7619 | 1 | 1 | 2.35E-05 | 1E-05 | 0.00142 | 0.002 |
| CETINA | 0.004181 |  | 1 | 3.701E-05 | 0.001553 | 1 | 0.01413 | 1 | 1 |
| BUDOŠKE BARE | 0.7619 | 1 |  | 0.01927 | 0.7324 | 0.009554 | 0.00026 | 0.6021 | 1 |
| VRANA LAKE | 1 | 3.701E-05 | 0.01927 |  | 1 | 4.78E-07 | 2.1E-07 | 1E-05 | 9E-06 |
| NIN | 1 | 0.001553 | 0.7324 | 1 |  | 3.73E-06 | 9.1E-07 | 0.00027 | 0.0003 |
| PAG | 2.35E-05 | 1 | 0.009554 | 4.775E-07 | 0.000003726 |  | 0.9649 | 1 | 1 |
| SKADAR LAKE | 1E-05 | 0.01413 | 0.000261 | 2.089E-07 | 9.085E-07 | 0.9649 |  | 0.7455 | 0.0772 |
| PANDURICA | 0.001416 | 1 | 0.6021 | 1.006E-05 | 0.0002744 | 1 | 0.7455 |  | 1 |
| LOVĆEN | 0.002024 | 1 | 1 | 8.607E-06 | 0.0003147 | 1 | 0.07717 | 1 |  |
