# Supplementary figures and images for: Uncovering the genomic basis of phenological traits in Chouardia litardierei (Asparagaceae) through a genome-wide association study (GWAS)
Source: Front Plant Sci. 2025 Apr 17;16:1571608. doi: 10.3389/fpls.2025.1571608 (PMC12070586; doi:10.3389/fpls.2025.1571608)

**
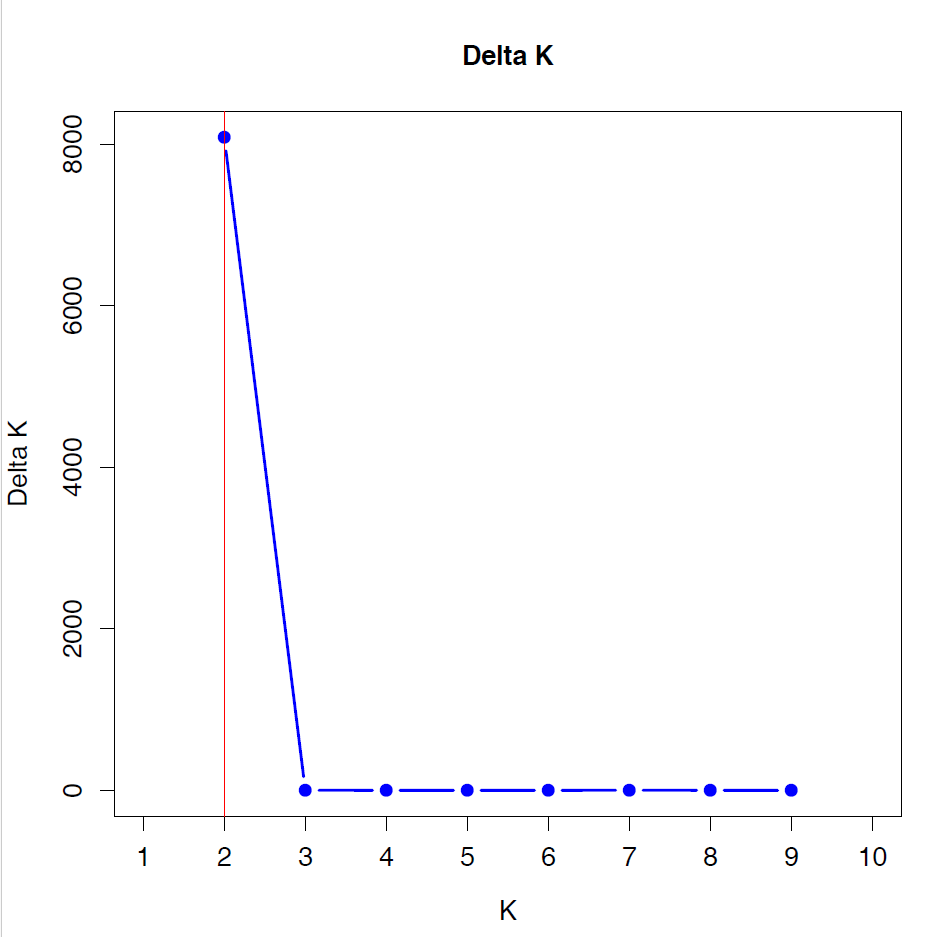
Figure 1.** Delta K values as obtained by the STRUCTURE software.

Supplement: Supplementary file 3 [file Table3.docx]
