# Supplementary material for: Uncovering the genomic basis of phenological traits in Chouardia litardierei (Asparagaceae) through a genome-wide association study (GWAS)
Source: Front Plant Sci. 2025 Apr 17;16:1571608. doi: 10.3389/fpls.2025.1571608 (PMC12070586; doi:10.3389/fpls.2025.1571608)
Supplement: Supplementary file 4 [file Table4.docx]

**
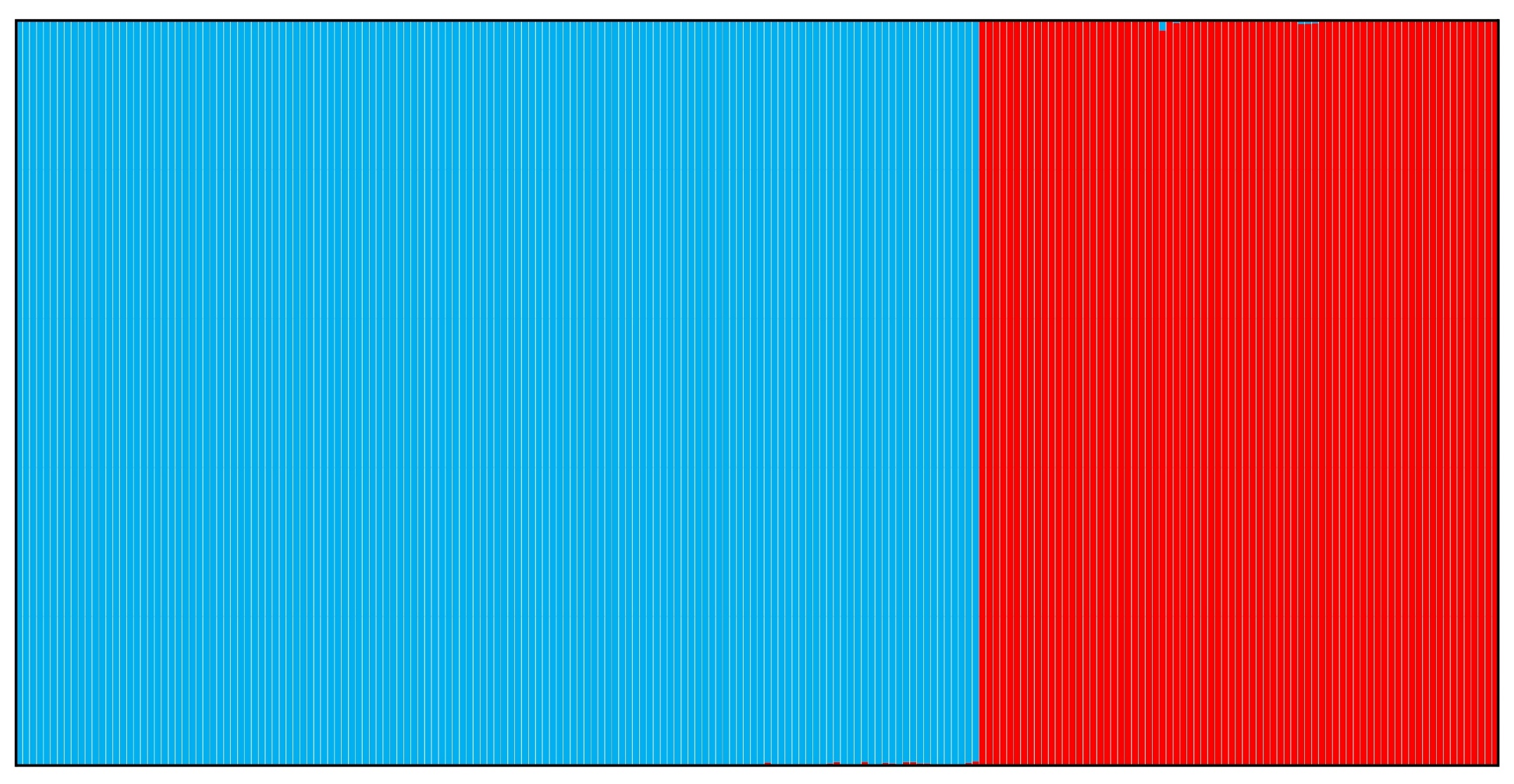
Figure 1.** Population-genetic structure of studied *Chouardia* *litardierei* populations as revealed by the STRUCTURE software. Each stacked column represents a single individual. Individuals belonging to the meadow and the seashore groups of populations are marked with blue, and individuals from the dolomite habitat populations are marked with red.
