# Supplementary material for: Uncovering the genomic basis of phenological traits in Chouardia litardierei (Asparagaceae) through a genome-wide association study (GWAS)
Source: Front Plant Sci. 2025 Apr 17;16:1571608. doi: 10.3389/fpls.2025.1571608 (PMC12070586; doi:10.3389/fpls.2025.1571608)
Supplement: Supplementary file 5 [file Table5.docx]

**Table 1.** SNPs identified as having a major sparse effect (PIP > 0.1) on **FPD**, **VPD**, **BPF** and **BOS** traits in the multi-SNP Bayesian sparse linear mixed model (BSLMM) analysis.

| Trait | **SNP** | **Chr** | **Position** | **Multi-SNP BSLMM Analysis *β* (PIP)** |
| --- | --- | --- | --- | --- |
| FPD | 131957_13 | 10 | 97222552 | -0.699 (0.167) |
|  | 750129_37 | 7 | 113120650 | -0.476 (0.165) |
| VPD | 565532_39 | 4 | 14626431 | -0.327 (0.912) |
|  | 210123_39 | 12 | 239066297 | 0.328 (0.748) |
|  | 64746_61 | 9 | 23100056 | 0.222 (0.579) |
|  | 608473_26 | 4 | 312243109 | -0.228 (0.447) |
|  | 169192_99 | 11 | 71192707 | -0.212 (0.393) |
|  | 165641_27 | 11 | 59678762 | -0.224 (0.308) |
|  | 22755_41 | 8 | 27370914 | -0.229 (0.286) |
|  | 392671_80 | 13 | 668483795 | 0.188 (0.277) |
|  | 345151_44 | 13 | 477215139 | 0.159 (0.236) |
|  | 68440_39 | 9 | 3481745 | -0.295 (0.232) |
|  | 114136_23 | 10 | 203465059 | -0.244 (0.225) |
|  | 131957_13 | 10 | 97222552 | 0.181 (0.224) |
|  | 158923_36 | 11 | 31022405 | -0.174 (0.224) |
|  | 651864_26 | 5 | 14713180 | -0.161 (0.192) |
|  | 16906_26 | 8 | 164769480 | -0.213 (0.188) |
|  | 274011_25 | 13 | 193652215 | -0.183 (0.184) |
|  | 203695_51 | 12 | 214471601 | -0.161 (0.180) |
|  | 562222_42 | 4 | 133794937 | -0.135 (0.175) |
|  | 206941_26 | 12 | 227677762 | -0.206 (0.175) |
|  | 431951_18 | 13 | 86851653 | 0.173 (0.167) |
|  | 518753_26 | 3 | 131097306 | -0.206 (0.164) |
|  | 404599_18 | 13 | 718065214 | 0.176 (0.158) |
|  | 264719_36 | 13 | 154558733 | 0.211 (0.152) |
|  | 345740_18 | 13 | 478800231 | -0.186 (0.152) |
|  | 169723_49 | 11 | 73174727 | 0.177 (0.148) |
|  | 455977_31 | 1 | 38506814 | -0.187 (0.146) |
|  | 779448_31 | 7 | 37409277 | -0.127 (0.141) |
|  | 453245_41 | 1 | 28792186 | 0.184 (0.141) |
|  | 175156_81 | 11 | 97958069 | -0.176 (0.139) |
|  | 732487_30 | 6 | 50042305 | -0.172 (0.137) |
|  | 236070_45 | 12 | 39684927 | -0.245 (0.136) |
|  | 794077_90 | 7 | 99621809 | 0.159 (0.135) |
|  | 713226_25 | 6 | 119601819 | 0.135 (0.131) |
|  | 167443_28 | 11 | 6497513 | 0.177 (0.129) |
|  | 628242_25 | 4 | 62348852 | 0.144 (0.122) |
|  | 708419_55 | 6 | 102061527 | -0.147 (0.122) |
|  | 108954_31 | 10 | 185953028 | -0.140 (0.119) |
|  | 56223_19 | 9 | 159032132 | 0.169 (0.119) |
|  | 168723_26 | 11 | 69545761 | 0.165 (0.117) |
|  | 321566_29 | 13 | 384493611 | 0.190 (0.115) |
|  | 571637_57 | 4 | 172299280 | -0.166 (0.115) |
|  | 63780_22 | 9 | 20268889 | 0.150 (0.115) |
|  | 144881_40 | 11 | 149677564 | 0.149 (0.115) |
|  | 196140_32 | 12 | 183140101 | -0.194 (0.114) |
|  | 41769_13 | 9 | 100304229 | 0.123 (0.114) |
|  | 243670_21 | 12 | 66893770 | -0.094 (0.114) |
|  | 446954_131 | 1 | 137876700 | 0.195 (0.111) |
|  | 625480_18 | 4 | 5030882 | -0.155 (0.111) |
|  | 293282_20 | 13 | 27101492 | -0.144 (0.109) |
|  | 760751_41 | 7 | 159527948 | -0.123 (0.109) |
|  | 338697_27 | 13 | 45325469 | -0.148 (0.108) |
|  | 597865_16 | 4 | 273124373 | -0.140 (0.107) |
|  | 633475_14 | 4 | 84524636 | 0.130 (0.107) |
|  | 790359_35 | 7 | 80015952 | -0.128 (0.107) |
|  | 584545_89 | 4 | 22256369 | 0.115 (0.106) |
|  | 76972_41 | 9 | 66872924 | 0.164 (0.106) |
|  | 278704_14 | 13 | 212393830 | 0.127 (0.104) |
|  | 723031_44 | 6 | 159155591 | -0.119 (0.103) |
|  | 207532_23 | 12 | 229820496 | 0.106 (0.102) |
|  | 173326_23 | 11 | 91514076 | -0.124 (0.102) |
|  | 577335_20 | 4 | 194281453 | -0.129 (0.101) |
|  | 513067_18 | 3 | 110714483 | 0.098 (0.100) |
|  | 296028_31 | 13 | 282176585 | 0.169 (0.100) |
|  | 346507_22 | 13 | 481318344 | 0.092 (0.100) |
|  | 321869_23 | 13 | 385455641 | 0.131 (0.099) |
|  | 318805_22 | 13 | 374121017 | -0.189 (0.099) |
|  | 541757_17 | 3 | 63012003 | 0.113 (0.099) |
|  | 373292_20 | 13 | 588262669 | 0.133 (0.099) |
|  | 541107_21 | 3 | 59731100 | -0.211 (0.098) |
|  | 369203_29 | 13 | 572629490 | 0.096 (0.098) |
|  | 642210_29 | 5 | 103184092 | -0.132 (0.097) |
|  | 486980_18 | 2 | 22661955 | 0.116 (0.096) |
|  | 162069_19 | 11 | 45040054 | 0.135 (0.096) |
|  | 634062_21 | 4 | 8802327 | 0.098 (0.096) |
|  | 171198_47 | 11 | 79969607 | -0.140 (0.095) |
| BOF | 504422_54 | 2 | 95535920 | 0.321 (0.172) |
|  | 337862_27 | 13 | 450832737 | 0.281 (0.156) |
|  | 633306_18 | 4 | 83549086 | -0.378 (0.098) |
| BOS | 565532_39 | 4 | 14626431 | 0.437 (0.959) |
|  | 210123_39 | 12 | 239066297 | -0.482 (0.829) |
|  | 723031_44 | 6 | 159155591 | 0.307 (0.356) |
|  | 175156_81 | 11 | 97958069 | 0.342 (0.277) |
|  | 114136_23 | 10 | 203465059 | 0.376 (0.241) |
|  | 64746_61 | 9 | 23100056 | 0.229 (0.222) |
|  | 203695_51 | 12 | 214471601 | 0.279 (0.221) |
|  | 165641_27 | 11 | 59678762 | 0.310 (0.219) |
|  | 206941_26 | 12 | 227677762 | 0.391 (0.208) |
|  | 571637_57 | 4 | 172299280 | 0.353 (0.202) |
|  | 169192_99 | 11 | 71192707 | 0.245 (0.177) |
|  | 131957_13 | 10 | 97222552 | -0.286 (0.172) |
|  | 121534_19 | 10 | 48645623 | 0.326 (0.169) |
|  | 455977_31 | 1 | 38506814 | 0.358 (0.156) |
|  | 252718_24 | 13 | 10389007 | 0.208 (0.143) |
|  | 441735_22 | 1 | 121138696 | -0.292 (0.138) |
|  | 345740_18 | 13 | 478800231 | 0.284 (0.133) |
|  | 391526_58 | 13 | 661633656 | 0.300 (0.126) |
|  | 708419_55 | 6 | 102061527 | 0.239 (0.123) |
|  | 651208_30 | 5 | 143900923 | -0.177 (0.123) |
|  | 732487_30 | 6 | 50042305 | 0.358 (0.121) |
|  | 392671_80 | 13 | 668483795 | -0.236 (0.117) |
|  | 657278_42 | 5 | 171253039 | 0.193 (0.103) |
|  | 518753_26 | 3 | 131097306 | 0.264 (0.100) |
|  | 437888_20 | 1 | 105737657 | 0.228 (0.096) |
|  | 496686_32 | 2 | 61419316 | -0.311 (0.095) |
|  | 565532_39 | 4 | 14626431 | 0.437 (0.959) |
|  | 210123_39 | 12 | 239066297 | -0.482 (0.829) |
|  | 723031_44 | 6 | 159155591 | 0.307 (0.356) |
|  | 175156_81 | 11 | 97958069 | 0.342 (0.277) |
|  | 114136_23 | 10 | 203465059 | 0.376 (0.241) |
|  | 64746_61 | 9 | 23100056 | 0.229 (0.222) |
|  | 203695_51 | 12 | 214471601 | 0.279 (0.221) |
|  | 165641_27 | 11 | 59678762 | 0.310 (0.219) |
|  | 206941_26 | 12 | 227677762 | 0.391 (0.208) |
|  | 571637_57 | 4 | 172299280 | 0.353 (0.202) |
|  | 169192_99 | 11 | 71192707 | 0.245 (0.177) |
|  | 131957_13 | 10 | 97222552 | -0.286 (0.172) |
|  | 121534_19 | 10 | 48645623 | 0.326 (0.169) |
|  | 455977_31 | 1 | 38506814 | 0.358 (0.156) |
|  | 252718_24 | 13 | 10389007 | 0.208 (0.143) |
|  | 441735_22 | 1 | 121138696 | -0.292 (0.138) |
|  | 345740_18 | 13 | 478800231 | 0.284 (0.133) |
|  | 391526_58 | 13 | 661633656 | 0.300 (0.126) |
|  | 708419_55 | 6 | 102061527 | 0.239 (0.123) |
|  | 651208_30 | 5 | 143900923 | -0.177 (0.123) |
|  | 732487_30 | 6 | 50042305 | 0.358 (0.121) |
|  | 392671_80 | 13 | 668483795 | -0.236 (0.117) |
|  | 657278_42 | 5 | 171253039 | 0.193 (0.103) |
|  | 518753_26 | 3 | 131097306 | 0.264 (0.100) |
|  | 437888_20 | 1 | 105737657 | 0.228 (0.096) |
|  | 496686_32 | 2 | 61419316 | -0.311 (0.095) |

BSLMM was fitted on 23,315 SNPs.; BSLMM, Bayesian sparse linear mixed model; BOF, Beginning of Flowering; BOS, Beginning of Sprouting; Chr, Chromosome; FPD, Flowering Period Duration; PIP, Posterior Inclusion Probability; SNP, Single Nucleotide Polymorphism; VPD, Vegetation Period Duration.
