# Supplementary material for: Uncovering the genomic basis of phenological traits in Chouardia litardierei (Asparagaceae) through a genome-wide association study (GWAS)
Source: Front Plant Sci. 2025 Apr 17;16:1571608. doi: 10.3389/fpls.2025.1571608 (PMC12070586; doi:10.3389/fpls.2025.1571608)
Supplement: Supplementary file 6 [file Table6.docx]

**
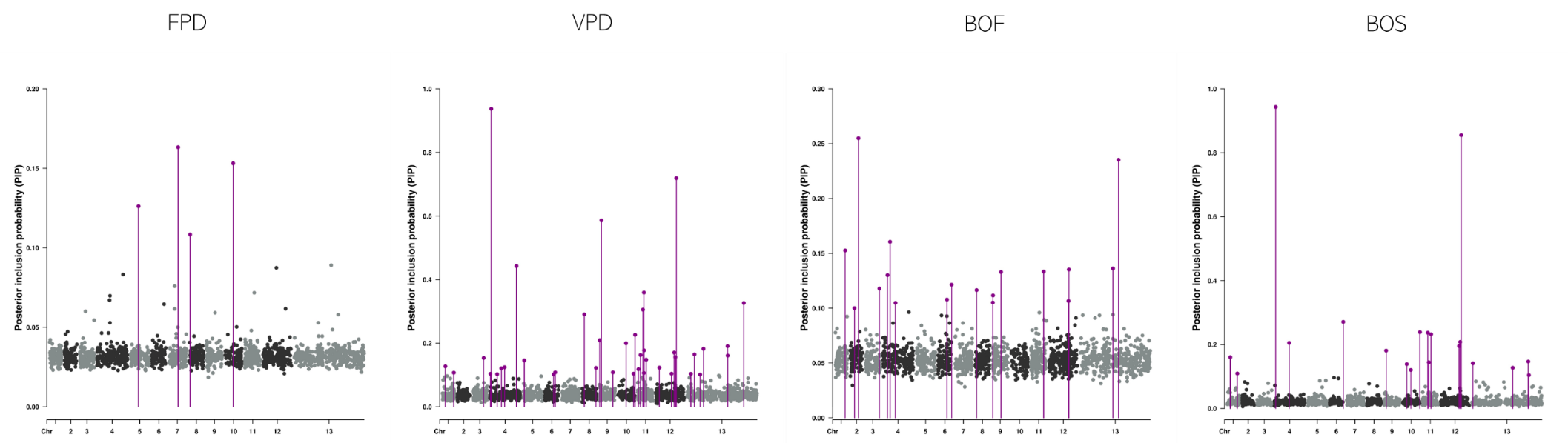
Figure 1.** Manhattan plots of the BSLMM analysis for the FPD, VPD, BOF, and BOS traits of the *Chouardia litardierei*. The x-axis represents the chromosomal position of SNPs, and the y-axis represents their posterior inclusion probabilities (PIPs).

BSLMM; Bayesian Sparse Linear Mixed Model, BOF, Beginning of Flowering; BOS, Beginning of Sprouting; FPD, Flowering Period Duration; VPD, Vegetation Period Duration.
