# Supplementary material for: Uncovering the genomic basis of phenological traits in Chouardia litardierei (Asparagaceae) through a genome-wide association study (GWAS)
Source: Front Plant Sci. 2025 Apr 17;16:1571608. doi: 10.3389/fpls.2025.1571608 (PMC12070586; doi:10.3389/fpls.2025.1571608)
Supplement: Supplementary file 7 [file Table7.docx]

**Table 1.** Means, medians, and 95% equal tail posterior probability intervals (95% ETPPIs) of hyperparameters estimated from the Bayesian sparse linear mixed model (BSLMM) in phenological trait **FPD**, **VPD**, **BOF** and **BOS**.

| Trait | **Hyperparameter** | **Mean** | **Median** | **2.5%** | **97.5%** |
| --- | --- | --- | --- | --- | --- |
| FPD | h | 0.3244 | 0.3128 | 0.0316 | 0.6830 |
|  | PVE | 0.2026 | 0.1798 | 0.0171 | 0.5215 |
|  | rho | 0.5220 | 0.5315 | 0.0275 | 0.9799 |
|  | PGE | 0.4722 | 0.4776 | - | 0.9671 |
|  | pi | 3.23 × 10^-2^ | 1.06 × 10^-2^ | 6.40 × 10^-4^ | 1.42 × 10^-1^ |
|  | n.gamma | 60.67 | 20.00 | - | 267.00 |
| VPD | h | 0.8224 | 0.8305 | 0.6667 | 0.9312 |
|  | PVE | 0.8695 | 0.8717 | 0.7803 | 0.9451 |
|  | rho | 0.6511 | 0.6902 | 0.1185 | 0.9872 |
|  | PGE | 0.6572 | 0.7720 | 0.0553 | 0.9947 |
|  | pi | 5.86 × 10^-2^ | 4.62 × 10^-2^ | 2.18 × 10^-3^ | 1.55 × 10^-1^ |
|  | n.gamma | 111.28 | 88.00 | 4.00 | 290.00 |
| BOF | h | 0.7046 | 0.7164 | 0.4483 | 0.8934 |
|  | PVE | 0.6603 | 0.6622 | 0.4659 | 0.8455 |
|  | rho | 0.3267 | 0.2735 | 0.0118 | 0.8882 |
|  | PGE | 0.2586 | 0.1556 | - | 0.9121 |
|  | pi | 2.52 × 10^-2^ | 1.07 × 10^-2^ | 6.14 × 10^-4^ | 1.11 × 10^-1^ |
|  | n.gamma | 47.29 | 20.00 | 0.00 | 207.00 |
| BOS | h | 0.7319 | 0.7414 | 0.5371 | 0.8738 |
|  | PVE | 0.7605 | 0.7616 | 0.6332 | 0.8829 |
|  | rho | 0.6346 | 0.6475 | 0.2029 | 0.9796 |
|  | PGE | 0.6319 | 0.6870 | 0.1598 | 0.9888 |
|  | pi | 3.05 × 10^-2^ | 2.25 × 10^-2^ | 2.87 × 10^-3^ | 1.09 × 10^-1^ |
|  | n.gamma | 52.58 | 39.00 | 5.00 | 189.00 |

BSLMM was fitted on 23,315 SNPs. BOF, Beginning of Flowering; BOS, Beginning of Sprouting; FPD, Flowering Period Duration; h, approximation to the proportion of phenotypic variance explained by variants; n.gamma, number of variants with major effect; PGE, Proportion of Genetic variance explained by variants with major effect; pi, proportion of variants with non-zero effects; PVE, proportion of phenotypic variance explained by variants; rho, approximation to the proportion of genetic variance explained by variants with major effect; VPD, Vegetation Period Duration.
