# Supplementary material for: Uncovering the genomic basis of phenological traits in Chouardia litardierei (Asparagaceae) through a genome-wide association study (GWAS)
Source: Front Plant Sci. 2025 Apr 17;16:1571608. doi: 10.3389/fpls.2025.1571608 (PMC12070586; doi:10.3389/fpls.2025.1571608)
Supplement: Supplementary file 8 [file Table8.docx]

**Table 1.** SNPs passing the genome-wide significance threshold (*p* < 1 × 10⁻³) in the multivariate linear mixed model (mvLMM) analysis for VPD and BOS traits of *Chouardia litardierei* in GEMMA multivariate GWAS.

| SNP | **Chr** | **Position** | **Effect**  **allele** | **Reference**  **allele** | **Beta1 (VPD)** | **Beta2 (BOS)** | **mvLMM Analysis in GEMMA  (*p*-value)** |
| --- | --- | --- | --- | --- | --- | --- | --- |
| 475214_20 | 2 | 10692011 | T | C | 0.01 | -0.16 | 6.68 × 10⁻¹⁰ |
| 654470_57 | 5 | 159590069 | G | A | -0.81 | 0.23 | 9.18 × 10⁻¹⁰ |
| 689821_13 | 5 | 93776308 | A | C | -0.77 | 0.22 | 1.66 × 10⁻⁸ |
| 83332_22 | 9 | 9096582 | G | A | 0.18 | -0.31 | 4.37 × 10⁻⁸ |
| 558534_14 | 4 | 116336173 | T | A | -0.92 | 0.48 | 6.36 × 10⁻⁸ |
| 145174_73 | 11 | 15061603 | C | G | -0.54 | -0.01 | 1.02 × 10⁻⁷ |
| 522007_32 | 3 | 141694985 | C | T | -0.32 | -0.07 | 2.72 × 10⁻⁷ |
| 179782_120 | 12 | 117670370 | T | C | -0.49 | 0.02 | 3.20 × 10⁻⁷ |
| 248115_31 | 12 | 8479581 | A | T | -0.51 | -0.06 | 3.46 × 10⁻⁷ |
| 28256_68 | 8 | 50959986 | A | G | -0.38 | 0.67 | 4.19 × 10⁻⁷ |
| 187632_54 | 12 | 151393801 | T | G | -0.40 | -0.01 | 5.14 × 10⁻⁷ |
| 268970_17 | 13 | 171155860 | A | G | -0.49 | -0.03 | 7.85 × 10⁻⁷ |
| 307326_19 | 13 | 326551046 | A | G | -0.66 | 0.06 | 1.17 × 10⁻⁶ |
| 550061_33 | 3 | 97695604 | A | T | -0.46 | 0.88 | 1.24 × 10⁻⁶ |
| 26676_33 | 8 | 4513527 | C | G | -0.23 | -0.11 | 2.32 × 10⁻⁶ |
| 187238_23 | 12 | 149770349 | T | C | -0.34 | -0.08 | 3.29 × 10⁻⁶ |
| 227369_31 | 12 | 307596886 | C | A | -0.16 | -0.03 | 4.09 × 10⁻⁶ |
| 305761_25 | 13 | 320423026 | T | G | 0.35 | -0.37 | 7.85 × 10⁻⁶ |
| 467066_106 | 1 | 91683912 | C | T | -0.55 | 0.07 | 9.29 × 10⁻⁶ |
| 770850_26 | 7 | 195082174 | C | T | -0.61 | 0.26 | 9.98 × 10⁻⁶ |
| 65720_38 | 9 | 26233589 | A | G | 0.61 | -0.59 | 1.24 × 10⁻⁵ |
| 565532_39 | 4 | 14626431 | C | A | -0.45 | 0.55 | 1.73 × 10⁻⁵ |
| 674245_25 | 5 | 2834749 | C | G | -0.30 | 0.03 | 1.75 × 10⁻⁵ |
| 67781_20 | 9 | 32778369 | T | A | -0.16 | -0.19 | 2.65 × 10⁻⁵ |
| 221833_73 | 12 | 284678317 | C | G | -0.23 | 0.30 | 3.20 × 10⁻⁵ |
| 214650_53 | 12 | 255323079 | A | G | -0.74 | 0.46 | 4.01 × 10⁻⁵ |
| 651220_77 | 5 | 143922570 | T | G | -0.32 | -0.10 | 4.46 × 10⁻⁵ |
| 365394_49 | 13 | 555889659 | C | T | -0.28 | 0.14 | 4.51 × 10⁻⁵ |
| 723031_44 | 6 | 159155591 | G | A | -0.27 | 0.52 | 4.78 × 10⁻⁵ |
| 202818_127 | 12 | 210719984 | C | T | -0.57 | 0.30 | 5.72 × 10⁻⁵ |
| 622051_16 | 4 | 357265730 | A | G | -0.31 | 0.81 | 6.32 × 10⁻⁵ |
| 592384_37 | 4 | 253302099 | T | C | -0.16 | -0.43 | 6.73 × 10⁻⁵ |
| 537684_52 | 3 | 44057957 | T | G | -0.16 | -0.06 | 8.02 × 10⁻⁵ |
| 310101_18 | 13 | 337885640 | T | C | -0.33 | -0.05 | 9.55 × 10⁻⁵ |
| 681559_42 | 5 | 58303261 | G | T | -0.39 | 0.19 | 9.72 × 10⁻⁵ |
| 334377_114 | 13 | 437172692 | C | A | 0.75 | -0.62 | 9.93 × 10⁻⁵ |
| 600093_25 | 4 | 281363631 | A | G | -0.63 | 0.19 | 1.42 × 10⁻⁴ |
| 261183_91 | 13 | 142406888 | T | G | 0.22 | -0.48 | 1.43 × 10⁻⁴ |
| 328618_13 | 13 | 414580639 | T | A | -0.16 | -0.18 | 1.53 × 10⁻⁴ |
| 422540_18 | 13 | 792932133 | A | G | -0.87 | 0.44 | 1.55 × 10⁻⁴ |
| 607415_46 | 4 | 308484921 | A | G | 0.22 | -0.36 | 1.56 × 10⁻⁴ |
| 32158_19 | 8 | 64100022 | T | A | -0.05 | -0.21 | 1.74 × 10⁻⁴ |
| 177171_18 | 12 | 105568874 | A | G | -0.73 | 0.60 | 1.87 × 10⁻⁴ |
| 475216_15 | 2 | 10692219 | T | G | -0.01 | -0.18 | 1.91 × 10⁻⁴ |
| 657020_14 | 5 | 169994000 | G | A | -0.27 | 0.02 | 1.93 × 10⁻⁴ |
| 104384_40 | 10 | 170660529 | G | A | -0.25 | 0.04 | 1.97 × 10⁻⁴ |
| 771754_39 | 7 | 197927864 | G | C | 0.03 | -0.20 | 2.00 × 10⁻⁴ |
| 301480_19 | 13 | 302007580 | A | G | -0.35 | 0.06 | 2.07 × 10⁻⁴ |
| 167223_27 | 11 | 64125165 | T | G | -0.46 | 0.49 | 2.14 × 10⁻⁴ |
| 688909_13 | 5 | 89858040 | A | C | -0.04 | -0.20 | 2.15 × 10⁻⁴ |
| 858479_18 | 13 | 78001418 | T | C | 0.36 | -0.20 | 2.22 × 10⁻⁴ |
| 143076_28 | 11 | 14367538 | T | C | -0.27 | -0.07 | 2.24 × 10⁻⁴ |
| 548241_67 | 3 | 88011092 | T | C | -0.25 | -0.09 | 2.37 × 10⁻⁴ |
| 583_81 | 8 | 102136191 | C | A | -0.24 | 0.58 | 2.38 × 10⁻⁴ |
| 196140_32 | 12 | 183140101 | A | T | -0.74 | 0.37 | 2.41 × 10⁻⁴ |
| 540381_39 | 3 | 5572300 | A | T | 0.14 | -0.24 | 3.13 × 10⁻⁴ |
| 614876_51 | 4 | 334032848 | C | T | -0.01 | -0.13 | 3.20 × 10⁻⁴ |
| 790473_18 | 7 | 8052404 | A | T | 0.85 | -1.13 | 3.34 × 10⁻⁴ |
| 740200_45 | 6 | 89087646 | C | A | -0.50 | 0.04 | 3.66 × 10⁻⁴ |
| 71327_30 | 12 | 43693525 | T | G | -0.41 | 0.02 | 3.67 × 10⁻⁴ |
| 612469_18 | 13 | 325349603 | A | C | 0.14 | 0.16 | 3.70 × 10⁻⁴ |
| 364317_22 | 9 | 551367550 | A | G | -0.70 | 0.29 | 4.26 × 10⁻⁴ |
| 210123_39 | 4 | 239066297 | T | G | 0.51 | -0.58 | 4.28 × 10⁻⁴ |
| 419317_22 | 13 | 780074412 | T | C | -0.39 | -0.09 | 4.49 × 10⁻⁴ |
| 202068_64 | 12 | 207629434 | T | C | -0.31 | -0.02 | 4.57 × 10⁻⁴ |
| 60130_20 | 9 | 17391855 | C | T | -0.32 | 0.73 | 4.58 × 10⁻⁴ |
| 478635_49 | 8 | 122304660 | A | C | -0.30 | 0.04 | 4.73 × 10⁻⁴ |
| 170834_35 | 13 | 774970 | G | A | -0.35 | 0.11 | 4.82 × 10⁻⁴ |
| 381580_38 | 5 | 619787450 | T | G | -0.27 | 0.06 | 4.97 × 10⁻⁴ |
| 154168_43 | 12 | 180827479 | C | A | -0.27 | 0.57 | 5.07 × 10⁻⁴ |
| 774777_66 | 3 | 206933711 | C | T | 0.57 | -0.72 | 5.10 × 10⁻⁴ |
| 548930_13 | 4 | 90980761 | T | G | -0.53 | 0.23 | 5.11 × 10⁻⁴ |
| 170124_137 | 13 | 74451579 | A | G | -0.67 | 0.27 | 5.17 × 10⁻⁴ |
| 711345_25 | 9 | 112404079 | C | A | 0.04 | 0.15 | 5.20 × 10⁻⁴ |
| 207337_22 | 12 | 229211403 | A | C | 0.59 | -0.14 | 5.22 × 10⁻⁴ |
| 439975_31 | 12 | 114209920 | A | G | 0.01 | -0.19 | 5.30 × 10⁻⁴ |
| 212201_44 | 4 | 246320665 | T | C | -0.04 | -0.10 | 5.46 × 10⁻⁴ |
| 631869_38 | 9 | 7683248 | T | G | 0.70 | -0.46 | 5.65 × 10⁻⁴ |
| 767107_28 | 9 | 18271185 | A | G | -0.30 | 0.02 | 5.72 × 10⁻⁴ |
| 55253_106 | 7 | 155581032 | G | A | -0.34 | 0.08 | 5.81 × 10⁻⁴ |
| 322826_18 | 12 | 390026768 | A | T | -0.42 | 0.18 | 5.86 × 10⁻⁴ |
| 165592_49 | 4 | 59562398 | A | G | 0.04 | 0.23 | 5.94 × 10⁻⁴ |
| 529999_34 | 7 | 1714447 | A | G | -0.35 | -0.02 | 5.97 × 10⁻⁴ |
| 264780_28 | 13 | 154888633 | T | A | 0.12 | -0.62 | 6.07 × 10⁻⁴ |
| 234677_87 | 7 | 34919398 | G | C | -0.19 | 0.42 | 6.15 × 10⁻⁴ |
| 201405_32 | 12 | 204415035 | A | T | -0.27 | -0.07 | 6.26 × 10⁻⁴ |
| 618657_20 | 7 | 345766799 | A | G | 0.93 | -1.05 | 6.71 × 10⁻⁴ |
| 76756_18 | 12 | 65979867 | C | A | -0.63 | 0.19 | 6.81 × 10⁻⁴ |
| 71567_24 | 13 | 44564317 | T | C | 0.22 | -0.02 | 6.89 × 10⁻⁴ |
| 765142_28 | 9 | 175841496 | A | C | -0.83 | 1.17 | 6.90 × 10⁻⁴ |
| 57078_21 | 5 | 163441584 | A | C | 0.22 | -0.21 | 6.98 × 10⁻⁴ |
| 484023_15 | 3 | 144655732 | G | A | -0.50 | 0.11 | 7.03 × 10⁻⁴ |
| 78404_13 | 5 | 72519187 | T | A | -0.57 | 0.29 | 7.06 × 10⁻⁴ |
| 76416_37 | 7 | 64503815 | A | G | 0.35 | -0.38 | 7.19 × 10⁻⁴ |
| 545555_90 | 3 | 78147943 | T | G | -0.14 | -0.17 | 7.21 × 10⁻⁴ |
| 512981_36 | 12 | 110347767 | G | T | 0.06 | 0.16 | 7.26 × 10⁻⁴ |
| 684715_22 | 7 | 7273425 | T | C | -0.34 | 0.49 | 7.38 × 10⁻⁴ |
| 170122_38 | 12 | 74451257 | A | G | -0.66 | 0.26 | 7.50 × 10⁻⁴ |
| 200956_103 | 9 | 202665910 | C | T | 0.20 | 0.22 | 7.55 × 10⁻⁴ |
| 528481_19 | 12 | 165845777 | T | C | 0.74 | -0.55 | 7.72 × 10⁻⁴ |
| 180221_31 | 5 | 11898341 | C | A | -0.29 | 0.48 | 7.78 × 10⁻⁴ |
| 635043_17 | 4 | 93373391 | T | C | 0.37 | -0.44 | 8.20 × 10⁻⁴ |
| 581314_17 | 13 | 210039723 | A | G | -0.12 | 0.42 | 8.55 × 10⁻⁴ |
| 371940_27 | 4 | 583317033 | T | G | -0.55 | 0.16 | 8.66 × 10⁻⁴ |
| 76460_82 | 9 | 64634814 | G | C | -0.28 | 0.15 | 8.71 × 10⁻⁴ |
| 560706_36 | 7 | 127372766 | A | C | -0.30 | -0.06 | 8.82 × 10⁻⁴ |
| 792151_44 | 9 | 90140260 | A | G | 0.11 | -0.63 | 9.00 × 10⁻⁴ |
| 772653_20 | 12 | 200562469 | C | T | -0.32 | 0.53 | 9.03 × 10⁻⁴ |
| 775046_20 | 2 | 207737647 | A | G | 0.19 | 0.22 | 9.09 × 10⁻⁴ |
| 151787_41 | 5 | 173804269 | C | G | -0.11 | -0.05 | 9.58 × 10⁻⁴ |
| 476624_22 | 5 | 112342852 | A | G | 0.24 | -0.06 | 9.68 × 10⁻⁴ |
| 415244_13 | 9 | 7620614 | A | G | 0.13 | -0.26 | 9.75 × 10⁻⁴ |
| 77325_18 | 4 | 68217631 | A | C | -0.49 | 0.80 | 9.94 × 10⁻⁴ |

mvLMM in GEMMA was fitted on 23,315 SNPs. BOS, Beginning Sprouting; Chr, Chromosome; VPD, Vegetation Period Duration; mvLMM, multivariate Linear Mixed Model; SNP, Single Nucleotide Polymorphism.
