# Supplementary material for: Uncovering the genomic basis of phenological traits in Chouardia litardierei (Asparagaceae) through a genome-wide association study (GWAS)
Source: Front Plant Sci. 2025 Apr 17;16:1571608. doi: 10.3389/fpls.2025.1571608 (PMC12070586; doi:10.3389/fpls.2025.1571608)
Supplement: Supplementary file 9 [file Table9.docx]

**Table 1.** SNPs passing the genome-wide significance threshold (*p* < 1 × 10⁻³) in the multivariate linear mixed model (mvLMM) analysis for VPD and BOF traits of *Chouardia litardierei* in GEMMA multivariate GWAS.

| SNP | **Chr** | **Position** | **Effect**  **allele** | **Reference**  **allele** | **Beta1 (VPD)** | **Beta2 (BOF)** | **mvLMM Analysis in GEMMA  (*p*-value)** |
| --- | --- | --- | --- | --- | --- | --- | --- |
| 65720_38 | 9 | 26233589 | A | G | 0.59 | 0.25 | 8.71 × 10⁻⁶ |
| 305761_25 | 13 | 320423026 | T | G | 0.34 | 0.01 | 2.04 × 10⁻⁵ |
| 504422_54 | 2 | 95535920 | T | G | 0.23 | 0.49 | 2.16 × 10⁻⁵ |
| 565532_39 | 4 | 14626431 | C | A | -0.45 | 0.15 | 3.26 × 10⁻⁵ |
| 334377_114 | 13 | 437172692 | C | A | 0.73 | 0.49 | 7.39 × 10⁻⁵ |
| 28256_68 | 8 | 50959986 | A | G | -0.39 | 0.44 | 8.58 × 10⁻⁵ |
| 104625_19 | 10 | 171714671 | C | A | 0.55 | 0.71 | 8.73 × 10⁻⁵ |
| 622077_29 | 4 | 357364641 | C | T | 0.32 | -0.62 | 9.61 × 10⁻⁵ |
| 252813_22 | 13 | 104630774 | C | G | 0.10 | -1.27 | 1.03 × 10⁻⁴ |
| 528481_19 | 3 | 165845777 | T | C | 0.73 | 0.80 | 1.03 × 10⁻⁴ |
| 757985_15 | 7 | 148582555 | C | T | 0.47 | -0.77 | 1.40 × 10⁻⁴ |
| 321869_23 | 13 | 385455641 | T | C | 0.36 | 0.79 | 1.51 × 10⁻⁴ |
| 207869_16 | 12 | 231068248 | T | A | 0.23 | 0.60 | 1.57 × 10⁻⁴ |
| 631869_38 | 4 | 7683248 | T | G | 0.70 | 0.80 | 1.87 × 10⁻⁴ |
| 774777_66 | 7 | 206933711 | C | T | 0.61 | -0.27 | 2.41 × 10⁻⁴ |
| 431930_95 | 13 | 86813045 | A | T | 0.21 | 0.37 | 2.68 × 10⁻⁴ |
| 167223_27 | 11 | 64125165 | T | G | -0.46 | 0.16 | 2.74 × 10⁻⁴ |
| 221833_73 | 12 | 284678317 | C | G | -0.22 | 0.11 | 3.14 × 10⁻⁴ |
| 186978_19 | 12 | 148693882 | A | C | -0.22 | 0.82 | 4.17 × 10⁻⁴ |
| 226324_53 | 12 | 303921633 | A | G | 0.55 | -0.66 | 4.24 × 10⁻⁴ |
| 86910_49 | 10 | 104918258 | T | C | 0.15 | -0.50 | 4.33 × 10⁻⁴ |
| 618657_20 | 4 | 345766799 | A | G | 0.95 | -0.28 | 4.52 × 10⁻⁴ |
| 445520_34 | 1 | 133744238 | A | G | 0.06 | 0.39 | 5.72 × 10⁻⁴ |
| 723279_19 | 6 | 159836366 | C | T | 0.62 | -0.54 | 5.95 × 10⁻⁴ |
| 623516_30 | 4 | 40486247 | T | G | 0.31 | -0.33 | 6.08 × 10⁻⁴ |
| 207870_38 | 12 | 231068565 | C | T | 0.21 | 0.53 | 6.11 × 10⁻⁴ |
| 558534_14 | 4 | 116336173 | T | A | -0.92 | 0.30 | 6.51 × 10⁻⁴ |
| 76416_37 | 9 | 64503815 | A | G | 0.35 | 0.06 | 6.51 × 10⁻⁴ |
| 65785_19 | 9 | 26407191 | G | A | -0.18 | -0.13 | 6.61 × 10⁻⁴ |
| 210123_39 | 12 | 239066297 | T | G | 0.50 | -0.01 | 6.84 × 10⁻⁴ |
| 41769_13 | 9 | 100304229 | A | G | 0.28 | 0.53 | 7.44 × 10⁻⁴ |
| 144881_40 | 11 | 149677564 | T | C | 0.42 | -0.81 | 8.05 × 10⁻⁴ |
| 177171_18 | 12 | 105568874 | A | G | -0.73 | 0.13 | 8.30 × 10⁻⁴ |
| 708427_23 | 6 | 102085089 | A | C | -0.24 | -0.43 | 8.38 × 10⁻⁴ |
| 422713_13 | 13 | 793523369 | G | A | -0.32 | -0.53 | 8.45 × 10⁻⁴ |
| 171277_71 | 11 | 80221934 | T | C | 0.12 | -0.63 | 9.85 × 10⁻⁴ |

mvLMM in GEMMA was fitted on 23,315 SNPs. BOF, Beginning of Flowering; Chr, Chromosome; FPD, Flowering Period Duration; mvLMM, multivariate Linear Mixed Model; SNP, Single Nucleotide Polymorphism.
