# Supplementary material for: Uncovering the genomic basis of phenological traits in Chouardia litardierei (Asparagaceae) through a genome-wide association study (GWAS)
Source: Front Plant Sci. 2025 Apr 17;16:1571608. doi: 10.3389/fpls.2025.1571608 (PMC12070586; doi:10.3389/fpls.2025.1571608)
Supplement: Supplementary file 10 [file Table10.docx]

**
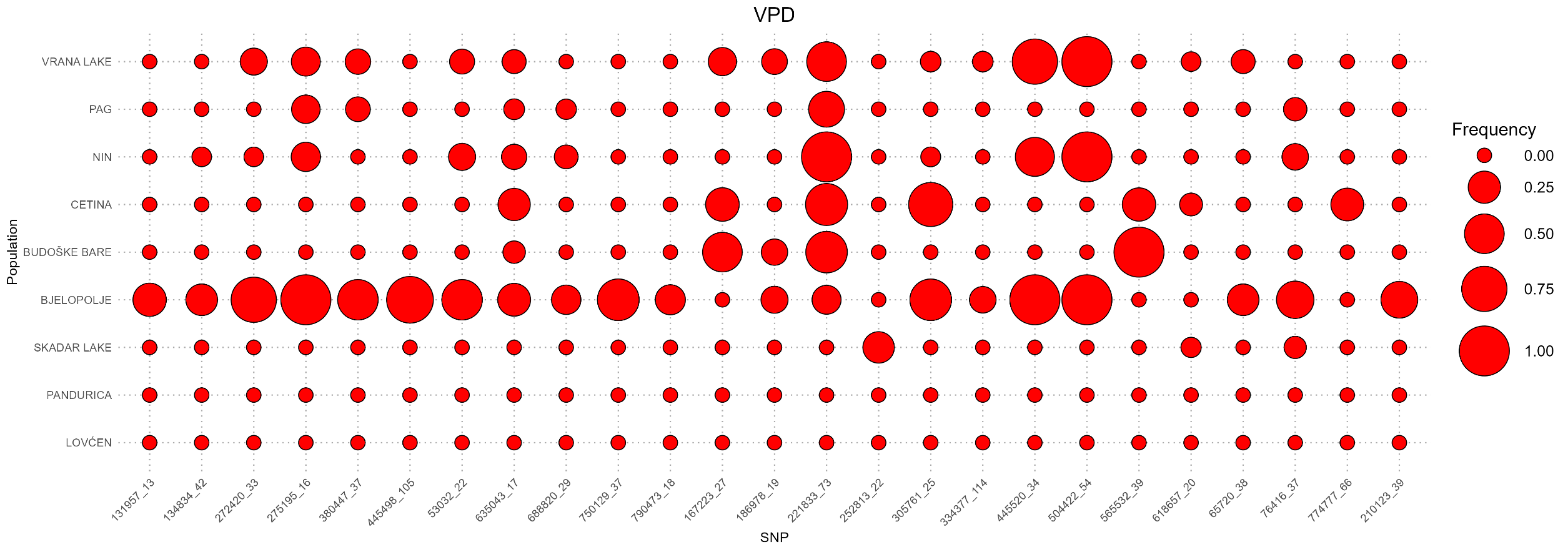
**

**Figure 1.** Frequency of effect alleles across populations for significant SNPs identified in the single-SNP LMM analysis (GEMMA and GMMAT), as well as the multi-SNP BSLMM analysis, all of which surpassed the genome-wide significance threshold (1 × 10⁻³) for the Vegetation Period Duration (VPD) trait. The analysis also includes SNPs meeting the same threshold in the multivariate GWAS. The corresponding SNPs are detailed in Table 3 and Table 5 of the manuscript.


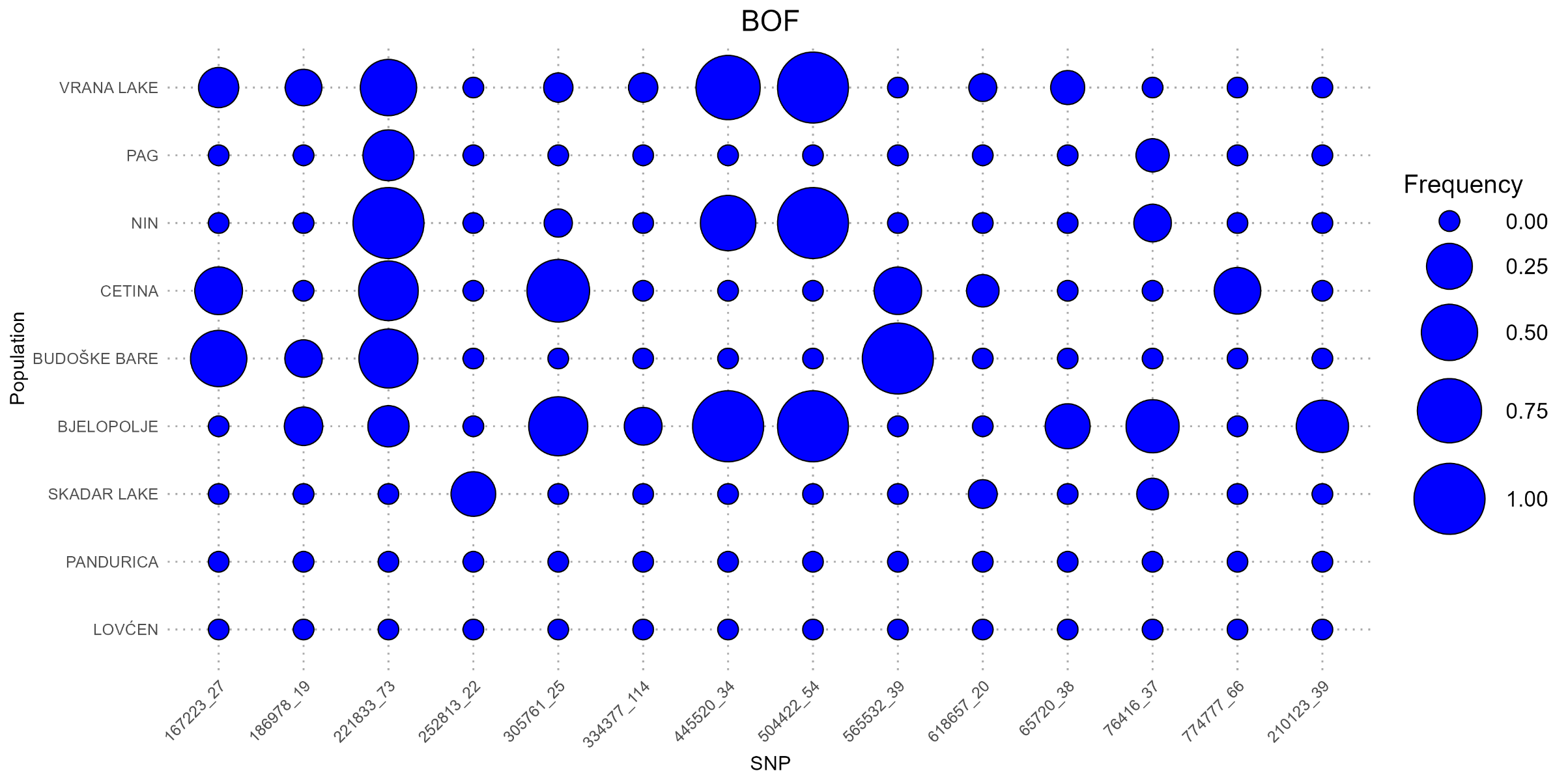
**Figure 2.** Frequency of effect alleles across populations for significant SNPs identified in the single-SNP LMM analysis (GEMMA and GMMAT), as well as the multi-SNP BSLMM analysis, all of which surpassed the genome-wide significance threshold (1 × 10⁻³) for the Beginning of Flowering (BOF) trait. The analysis also includes SNPs meeting the same threshold in the multivariate GWAS. The corresponding SNPs are detailed in Table 3 and Table 5 of the manuscript.


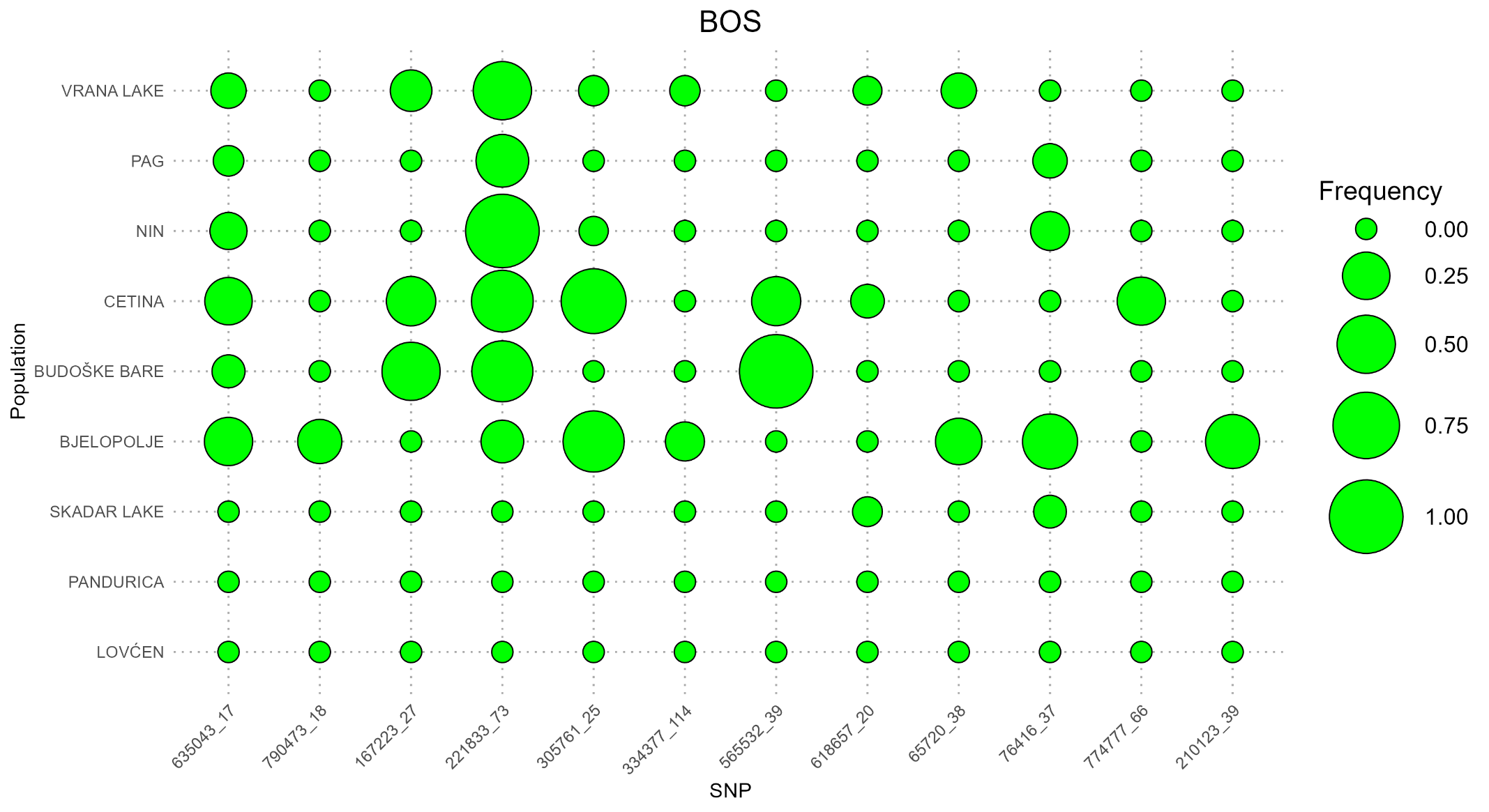
**Figure 3.** Frequency of effect alleles across populations for significant SNPs identified in the single-SNP LMM analysis (GEMMA and GMMAT), as well as the multi-SNP BSLMM analysis, all of which surpassed the genome-wide significance threshold (1 × 10⁻³) for the Beginning of Sprouting (BOS) trait. The analysis also includes SNPs meeting the same threshold in the multivariate GWAS. The corresponding SNPs are detailed in Table 3 and Table 5 of the manuscript.


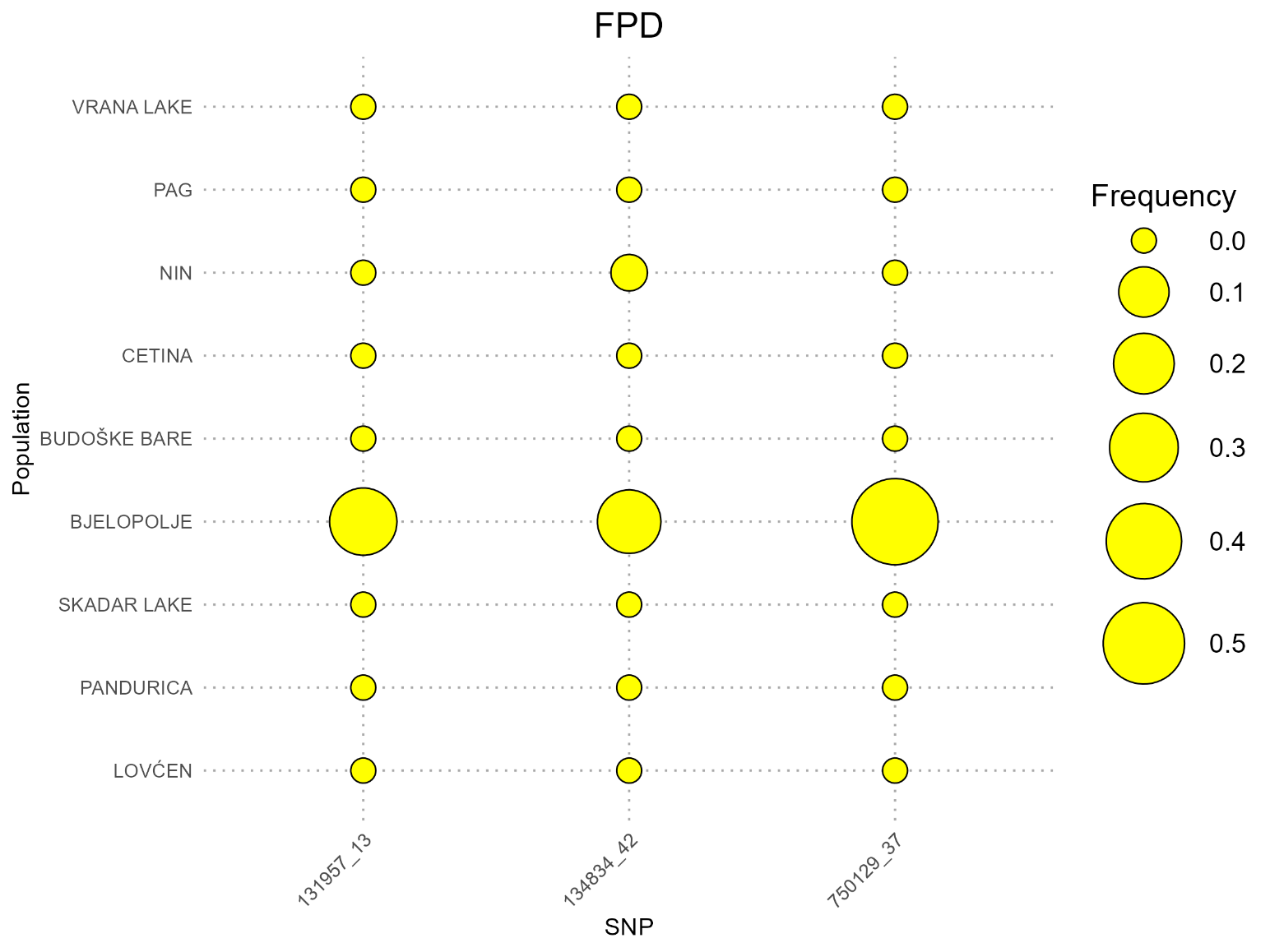


**Figure 4.** Frequency of effect alleles across populations for significant SNPs identified in the single-SNP LMM analysis (GEMMA and GMMAT), as well as the multi-SNP BSLMM analysis, all of which surpassed the genome-wide significance threshold (1 × 10⁻³) for the Flowering Period Duration (FPD) trait. The analysis also includes SNPs meeting the same threshold in the multivariate GWAS. The corresponding SNPs are detailed in Table 3 of the manuscript.
