# Supplementary material for: Uncovering the genomic basis of phenological traits in Chouardia litardierei (Asparagaceae) through a genome-wide association study (GWAS)
Source: Front Plant Sci. 2025 Apr 17;16:1571608. doi: 10.3389/fpls.2025.1571608 (PMC12070586; doi:10.3389/fpls.2025.1571608)

UNIVERSITY OF ZAGREB
FACULTY OF SCIENCE
Department of Biology
DIVISION OF BOTANY

Ivan Radosavljević
Division of Botany, Department of Biology,
Faculty of Science, University of Zagreb
Marulicev trg 9A
10000 Zagreb, Croatia
ivanrad@biol.pmf.hr
March 30th, 2025

Handling Editor

Rita Lourenço Costa

Frontiers in Plant Science

Dear Editor,

Thank you for the opportunity to revise our manuscript. We sincerely appreciate the valuable feedback you and the reviewers provided. We have carefully addressed each comment and revised the manuscript to enhance its clarity, accuracy, and overall quality. Please find our detailed responses below.

We appreciate your time and consideration and look forward to your feedback.

Best regards,
Ivan Radosavljević

Reviewer Comments:

Reviewer 1:

Dear Reviewer,

Before we go into detailed point-by-point replies, we thank the reviewer for the careful reading and constructive feedback. The comments helped us clarify key concepts, enhance the structure of the paper, and strengthen the arguments. We hope the replies are satisfactory and adequately address all concerns.

Main comments:

1. **GWAS studies often require very large sample sizes to identify reproducible SNP significant associations. In this work, a relatively small population of approximately 200 individuals was analyzed. This point should be included in the discussion, reflecting on its significance in relation to the outcomes.**

We thank the reviewer for the comment; the suggestion is appreciated and agreed upon. We acknowledge that GWAS studies typically require large sample sizes to detect robust SNP associations. In our study, the relatively small cohort of ~200 individuals represents a limitation. We have now included a statement in the Discussion section to address this point and to reflect on how the sample size may influence the strength and reproducibility of the findings. We also note that, despite the limited sample size, several biologically plausible associations were observed, which we believe warrant further investigation in larger cohorts. This limitation is now highlighted in the discussion, lines 587-592:

"The relatively small sample size is a limitation of our study, particularly given that GWAS typically include larger cohorts to detect robust and reproducible associations. Nevertheless, our analysis revealed several biologically plausible signals, which, while requiring validation, provide a valuable foundation for future studies. These findings should be interpreted with caution, but they offer meaningful insights that can be further explored and confirmed in larger, independent populations.".

2. **Phenotypic differences were found between populations and habitat groups. Was any experimental design taken into account in the common garden, e.g., blocks? Were any of these effects included in the statistical GWAS models?**

We thank the reviewer for the comment. We did not implement blocks in our common garden experiment, as the study area was very small (a few square meters) and we did not expect to observe any microenvironmental variation across the site. This homogeneity in environmental conditions made the use of blocking unnecessary. For a better understanding of the experiment, the following figure illustrates the experimental setup. Since all the plants were planted in the same soil mix, specially prepared for the purpose in one batch, and since no interventions of any kind were ever implemented (like watering, shading, or pest control), the only hypothetical variation across the experiment site could

originate from some bioclimatic element. However, considering the setup's dimensions, we believe that without any doubt, this possibility can be ruled out.

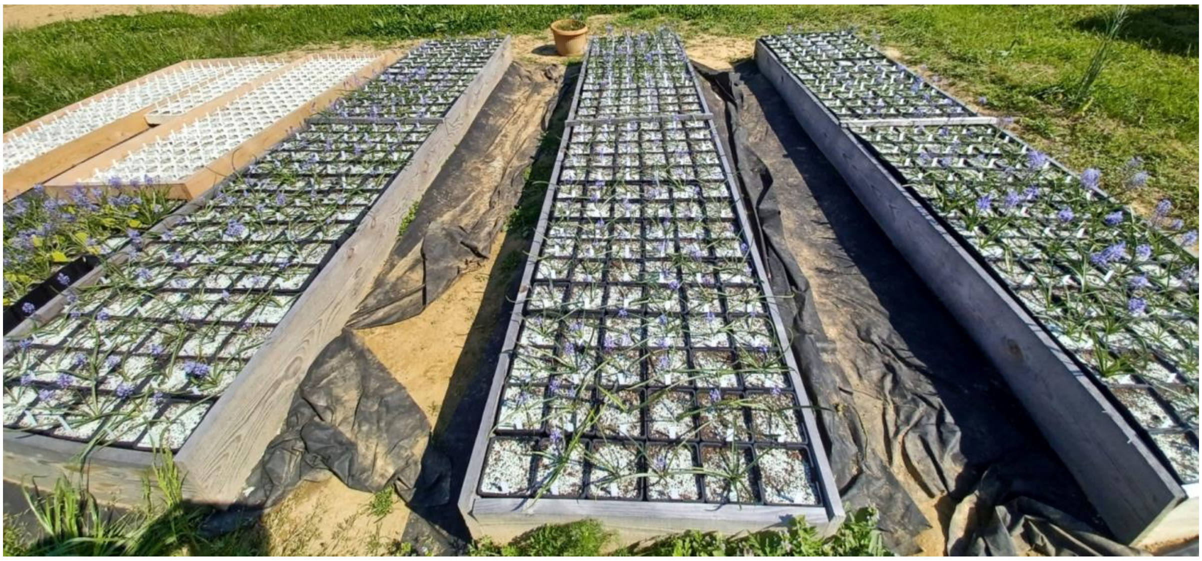

Since the block design was not implemented in the common garden experiment, block effects were not included in the GWAS models. However, population structure and relatedness were carefully addressed using multiple statistical approaches. In the frequentist framework, we applied linear mixed models (LMM) using GEMMA and Poisson generalized linear mixed models (GLMM) via GMMAT, both of which incorporate a genetic relatedness (kinship) matrix as a random effect to control for confounding due to shared ancestry. In the Bayesian framework, we employed a Bayesian sparse linear mixed model (BSLMM), which also incorporates a genetic relatedness (kinship) matrix as a random effect to account for population structure and relatedness while modelling both polygenic and sparse genetic effects. These complementary methods ensured that population stratification and relatedness were properly controlled for in the GWAS analyses, despite the absence of a block design.

3. **A detailed statistical explanation of the four GWAS models compared is provided. The different approaches result in a low number of common SNPs. What could explain these differing results? This point should be included in the discussion.**

We thank the reviewer for this comment and understand the concerns raised. The relatively low number of overlapping SNPs across the GWAS models can be attributed to differences in model assumptions and statistical frameworks. While the two frequentist approaches (LMM in GEMMA and Poisson GLMM in GMMAT) used a consistent significance threshold of $< 1 \times 10^{-3}$, the Bayesian approach (BSLMM) relies on posterior inclusion probabilities, which are conceptually different and often more conservative. These methodological differences likely contribute to the observed variation in detected SNPs. We have now addressed this point in the Discussion (lines 529-538) to clarify the implications for interpreting model-specific results:

"The relatively low overlap of significant SNPs detected across the different GWAS models likely reflects inherent differences in their statistical assumptions and approaches to modelling genetic effects. While both frequentist methods (GLMM and LMM) applied a consistent significance threshold of $< 1 \times 10^{-3}$, the BSLMM relies on posterior inclusion probabilities,

which are generally more conservative and not directly comparable to p-values. Importantly, each model is optimized for different data characteristics: LMM assumes normally distributed traits, whereas GLMM, using a Poisson distribution, is more appropriate for count-based traits with non-normal distributions. Applying trait-appropriate models increases the reliability and power of association detection, even if it results in a lower number of shared SNPs.".

4. **Please provide more information about the mvLMM model. For example, how does the mvLMM method account for population structure? Did you test a multi-trait model that included all four traits in the study? Not including FDP may bias the results—please clarify this point.**

We thank the reviewer for the comment. Multivariate GWAS was performed using the multivariate linear mixed model (mvLMM) implemented in GEMMA to investigate shared genetic associations between phenotypes. We have added this information to line 232. This model treats the traits as joint dependent variables and incorporates a genetic relatedness matrix to account for population structure and relatedness among individuals, which is described in lines 325–329.

We did not include all four traits simultaneously in the mvLMM. Instead, we selected trait pairs with the strongest phenotypic correlations (VPD–BOS and VPD–BOF) for multivariate analysis. This is because the power and interpretability of the mvLMM greatly depend on the degree of correlation between the traits. When traits are moderately to highly correlated, mvLMM is more effective at detecting shared genetic effects (e.g., due to pleiotropy), as it can borrow information across traits.

Including phenotypes with low or no correlation, such as FPD in our dataset, can reduce statistical power and obscure interpretation. In our case, FPD showed low correlation with the other traits and was therefore excluded from mvLMM analyses to maintain model efficiency and biological relevance.

Nonetheless, we emphasize that all traits, including FPD, were analyzed using univariate LMMs, ensuring no trait was omitted from association testing.

5. **In all GWAS studies, it is necessary to include a method to control for multiple testing, such as the Bonferroni correction or FDR adjustment. Why did you not apply any of them in your analysis? Please specify the ad hoc significance threshold applied.**

We appreciate the reviewer's comment regarding multiple testing correction. In our study, we applied an ad hoc genome-wide significance threshold of $p < 1\times10^{-3}$ for the frequentist GWAS analyses (GLMM, LMM, and mvLMM) (lines 236-237). The Bonferroni correction is often found to be too conservative, leading to very few significant SNP associations, and many studies have adopted less stringent thresholds to detect meaningful genetic signals [1–3]. In particular, a threshold of $p < 1\times10^{-3}$ has been used as a practical alternative in plant GWAS studies [4,5], especially given the variability in genome size, LD structure, and the effective number of independent tests across species.

While human GWAS often adopt the conventional 5×10^{-8} threshold—derived from a Bonferroni correction assuming ~1 million independent tests—such a universal threshold is less applicable in non-human systems.

Importantly, the choice of genome-wide threshold is conceptually linked more to prior probabilities and the expected number of true associations, rather than strictly to the number of tests performed in a given study. That is, the need for a stringent threshold arises not merely from the volume of testing, but from the low prior probability that any individual SNP is truly associated with the trait. This perspective justifies the use of conservative thresholds even when fewer tests are performed, and also supports a slightly more permissive threshold when the goal is to explore potentially meaningful signals for further validation.

Given the exploratory nature of our study and the moderate sample size, we selected a threshold that balances false discovery control with discovery potential. All candidate associations are interpreted cautiously, with the understanding that independent replication and/or functional validation are essential to confirm their relevance. We have now included this clarification in the revised manuscript in lines 587-592.

[1] Zhang H, Zhang J, Xu Q, Wang D, Di H, Huang J, et al. Identification of candidate tolerance genes to low-temperature during maize germination by GWAS and RNA-seqapproaches. BMC Plant Biol 2020;20:1–17. https://doi.org/10.1186/S12870-020-02543-9/FIGURES/6.
[2] Shi J, Wang Y, Wang C, Wang L, Zeng W, Han G, et al. Linkage mapping combined with GWAS revealed the genetic structural relationship and candidate genes of maize flowering time-related traits. BMC Plant Biol 2022;22:1–13. https://doi.org/10.1186/S12870-022-03711-9/FIGURES/5.
[3] Yang N, Lu Y, Yang X, Huang J, Zhou Y, Ali F, et al. Genome Wide Association Studies Using a New Nonparametric Model Reveal the Genetic Architecture of 17 Agronomic Traits in an Enlarged Maize Association Panel. PLoS Genet 2014;10:e1004573. https://doi.org/10.1371/JOURNAL.PGEN.1004573.
[4] Maldonado C, Mora F, Scapim CA, Coan M. Genome-wide haplotype-based association analysis of key traits of plant lodging and architecture of maize identifies major determinants for leaf angle: hapLA4. PLoS One 2019;14:e0212925. https://doi.org/10.1371/JOURNAL.PONE.0212925.
[5] Amalova A, Abugalieva S, Babkenov A, Babkenova S, Turuspekov Y. Genome-wide association study of yield components in spring wheat collection harvested under two water regimes in Northern Kazakhstan. PeerJ 2021;9:e11857. https://doi.org/10.7717/PEERJ.11857/SUPP-8.

6. **How are the 23,315 SNPs distributed across the chromosomes in relation to chromosome size? Chromosome 13 appears to contain more than twice the number of SNPs compared to the others (Figures 5 and 6).**

We thank the reviewer for the comment and appreciate the opportunity to clarify this point. In our previously published paper, where we presented the draft genome assembly of C. litardierei, we reported that Chromosome 13 is the largest of all chromosomes, measuring 825 Mbp in a genome of 3.7 Gbp, which accounts for almost a quarter of the total genomic information. Consequently, it is not a surprise to see that it contains by far the most SNPs when compared to other chromosomes. For more details, please see Figures 2 and 3, and Table 1 in our manuscript "Towards the Investigation of the Adaptive Divergence in a Species of Exceptional Ecological Plasticity: Chromosome-Scale Genome Assembly of Chouardia litardierei (Hyacinthaceae)".

Radosavljević, I., Križanović, K., Šarančić, S. L., & Jakše, J. (2023). Towards the Investigation of the Adaptive Divergence in a Species of Exceptional Ecological Plasticity: Chromosome-Scale Genome Assembly of Chouardia litardierei (Hyacinthaceae). International journal of molecular sciences, 24(13), 10755.

7. **How was population structure assessed using molecular markers? Including a PCA or Structure analysis would be helpful to better understand the genetic relationships between individuals and populations. This information is particularly relevant for interpreting the GWAS results.**

We thank the reviewer for the comment. Population structure and relatedness were addressed by calculating a full genomic relatedness (kinship) matrix using all 23,315 SNPs across individuals. This 23,315 × 23,315 matrix was incorporated as a random effect in all GWAS models (GEMMA, GMMAT, BSLMM, and mvLMM). As this matrix was included in the model to account for both population structure and cryptic relatedness, additional PCA or Structure analyses were not necessary.

However, we are aware that what you suggest is the presentation of a simpler, straightforward result representing the genetic structure of the studied populations. Since we have no objections to your suggestions, we have now employed the model-based clustering method, as implemented in the ParallelStructure software, for this purpose. Consequently, we made some minor additions to the manuscript in the Materials and methods, Results, and Discussion sections (lines 202-212, 365-370, and 472-476 respectively).

Minor comments:

Line 222: Add the software used to fit the mvLMM model.

We thank the reviewer for the suggestion. The software used to fit the mvLMM model is stated in line 232: "…multivariate linear mixed model (mvLMM) was perfomed in GEMMA…" and in line 323: "...using a multivariate linear mixed model (mvLMM) in GEMMA.".

Line 223: Correct the pairs of traits used for the mvLMM.

We thank the reviewer for the comment. The pairs of traits used in the mvLMM are correctly stated in the manuscript: multivariate GWAS was performed for VPD and BOS, and for VPD and BOF, based on their statistically significant correlations. We have reviewed the text and confirmed that this information is accurate and consistent. Please let us know if the concern refers to a different section or a specific inconsistency that may have been overlooked.

Line 257: The equation number for the LMM is incorrect. Move this sentence to the LMM section.

We thank the reviewer for pointing this out. You are correct - the equation number referenced in line 257 was incorrect and is now corrected in the revised version. However, the sentence itself must remain in the Bayesian framework section, as it refers to the standard LMM (Equation 4), which forms the basis of the BSLMM. Including this reference is essential for conceptual continuity, since the BSLMM extends the standard LMM by incorporating both

sparse and polygenic effects. We corrected the equation number and retained the sentence in its current location (line 268) for clarity.

Line 328: Remove this sentence.

We thank the reviewer for the comment. However, we cannot remove this sentence, as it is the only one providing a description of Figure 3. If preferred, we could consider placing it elsewhere in the manuscript to improve clarity. In the revised manuscript, this sentence is now placed in lines 341-342.

Line 343: These correlation results could be better presented in a figure or table to facilitate the visualization of positively and negatively correlated traits and to better understand the traits chosen for the multi-trait model.

We thank the reviewer for the suggestion. We have created Table 2 to present the correlation results, which facilitates the visualization of positively and negatively correlated traits. Corresponding text revisions have been added in lines 356-358.

Line 358: Use the model names instead of the software names when presenting results. Replace "GEMMA" with "LMM" and "GMMAT" with "Poisson GLMM".

We thank the reviewer for this very useful comment. We apologise for this oversight. We have now corrected this throughout the manuscript and we believe it now better represents our analytical approach.

Table 1 (by location) and Figure 4 present the same information. Consider retaining only one for clarity.

We thank the reviewer for the comment. We have removed part of Table 1 (by location) to avoid redundancy, and now only Figure 4 presents this information for clarity.

Reviewer 2:

Dear Reviewer,

Before we go into detailed point-by-point replies, thank you for all your comments and suggestions. We agree that perhaps some aspects of the manuscript can be further improved, and we hope you will find our replies satisfactory.

Comment:

This manuscript presents an insightful study on the phenological variation and genomic basis of traits in *C. litardierei*. The results are well-structured, and the statistical analyses are thorough, offering significant insights into the genetic underpinnings of flowering and fruiting traits in this species. The use of multiple GWAS tools and Bayesian approaches is commendable and strengthens the findings.

Suggestions:

The authors could consider performing further analyses, such as linkage disequilibrium (LD) analysis, to gain deeper insight into the relationship between SNPs. However, it is important to note that the effectiveness of such an analysis depends on the density of the SNPs captured by the sequencing method. If the SNP coverage is insufficient, the LD results may be affected, potentially leading to incomplete or misleading conclusions. Therefore, the authors may want to evaluate whether the current SNP density is adequate for meaningful LD analysis.

If a high-quality reference genome is available for this species, it would be valuable to incorporate SNP annotation to identify specific SNPs potentially affecting protein sequences. For example, focusing on missense mutations could offer a deeper understanding of the functional impacts of these variations.

We thank the reviewer for the thoughtful comments and suggestions. The genome of C. litardierei is approximately 3.7 Gbp, and our study used a reduced representation sequencing approach (ddRADseq), identifying 23,315 SNPs—resulting in a relatively low marker density of one SNP per 158 kbp. While sufficient for broad genomic patterns, ddRAD-seq inherently captures only a fraction of the genome, limiting SNP detection compared to whole-genome sequencing. This limitation is further compounded by the large genome size, making high-resolution assembly challenging. A higher-throughput genotyping approach could improve SNP resolution and annotation, particularly for functionally relevant variants like missense mutations.

Additionally, it is important to note that the reference genome for C. litardierei was only recently assembled for the first time. While it represents a valuable resource, it has not yet undergone the extensive refinements seen in model species, which have benefited from continuous improvements over many years or even decades. We recognize the potential benefits of SNP annotation and look forward to future research opportunities as more genomic resources become available for this species.

Regarding the LD analysis, we conducted an LD decay analysis, and the results indicated a slow LD decay, which is typical for wild species with less closely related individuals. A faster, more typical L-shaped LD decay curve is generally observed in populations of closely related individuals, such as crops or inbred lines of model species. The slow LD decay we observed aligns with the expected pattern for C. litardierei, considering its population structure. The threshold most commonly used for LD is 0.2, as marked in the attached figure. In our case, it corresponds to a distance of more than 1.1 M bases. From this perspective, the SNP density reached in this study appears sufficient for the purpose.

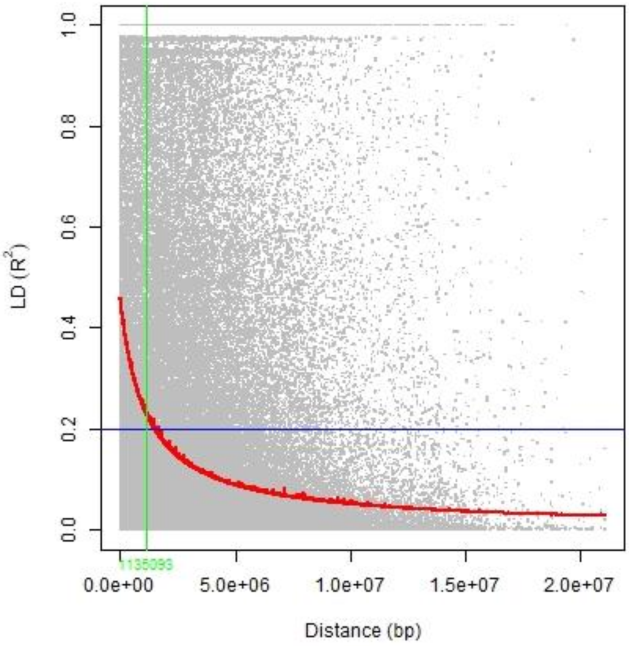

Supplement: Supplementary file 14 [file DataSheet1.pdf]
